# An epigenetic switch controls an alternative NR2F2 isoform that unleashes a metastatic program in melanoma

**DOI:** 10.1038/s41467-023-36967-2

**Published:** 2023-04-04

**Authors:** Veronica Davalos, Claudia D. Lovell, Richard Von Itter, Igor Dolgalev, Praveen Agrawal, Gillian Baptiste, David J. Kahler, Elena Sokolova, Sebastian Moran, Laia Piqué, Eleazar Vega-Saenz de Miera, Barbara Fontanals-Cirera, Alcida Karz, Aristotelis Tsirigos, Chi Yun, Farbod Darvishian, Heather C. Etchevers, Iman Osman, Manel Esteller, Markus Schober, Eva Hernando

**Affiliations:** 1 Department of Pathology, New York University Grossman School of Medicine, New York, NY 10016 USA; 2 Interdisciplinary Melanoma Cooperative Group, Perlmutter Cancer Center, New York University School of Medicine, New York, NY 10016 USA; 3 Josep Carreras Leukaemia Research Institute (IJC), Badalona, Barcelona, Catalonia Spain; 4 Applied Bioinformatics Laboratories, New York University Grossman School of Medicine, New York, NY 10016 USA; 5 Department of Molecular Pharmacology, Albert Einstein College of Medicine/ Montefiore, Bronx, NY 10461 USA; 6 High Throughput Biology Core, New York University Grossman School of Medicine, New York, NY 10016 USA; 7 The Ronald O. Perelman Department of Dermatology, New York University Grossman School of Medicine, New York, NY 10016 USA; 8 Aix-Marseille University, MMG, Inserm, U1251 Marseille, France; 9 Physiological Sciences Department, School of Medicine and Health Sciences, University of Barcelona (UB), Barcelona, Catalonia Spain; 10 Institució Catalana de Recerca i Estudis Avançats (ICREA), Barcelona, Catalonia Spain; 11 Centro de Investigacion Biomedica en Red, Cancer (CIBERONC), Madrid, Spain; 12 Department of Cell Biology, New York Grossman University School of Medicine, New York, NY 10016 USA

**Keywords:** DNA methylation, Melanoma

## Abstract

Metastatic melanoma develops once transformed melanocytic cells begin to de-differentiate into migratory and invasive melanoma cells with neural crest cell (NCC)-like and epithelial-to-mesenchymal transition (EMT)-like features. However, it is still unclear how transformed melanocytes assume a metastatic melanoma cell state. Here, we define DNA methylation changes that accompany metastatic progression in melanoma patients and discover Nuclear Receptor Subfamily 2 Group F, Member 2 – isoform 2 (NR2F2-Iso2) as an epigenetically regulated metastasis driver. *NR2F2*-Iso2 is transcribed from an alternative transcriptional start site (TSS) and it is truncated at the N-terminal end which encodes the NR2F2 DNA-binding domain. We find that NR2F2-Iso2 expression is turned off by DNA methylation when NCCs differentiate into melanocytes. Conversely, this process is reversed during metastatic melanoma progression, when *NR2F2*-Iso2 becomes increasingly hypomethylated and re-expressed. Our functional and molecular studies suggest that NR2F2-Iso2 drives metastatic melanoma progression by modulating the activity of full-length NR2F2 (Isoform 1) over EMT- and NCC-associated target genes. Our findings indicate that DNA methylation changes play a crucial role during metastatic melanoma progression, and their control of NR2F2 activity allows transformed melanocytes to acquire NCC-like and EMT-like features. This epigenetically regulated transcriptional plasticity facilitates cell state transitions and metastatic spread.

## Abstract (web summary)

Melanocytes can de-differentiate into neural crest cell (NCC)- like states during metastatic melanoma progression. Here the authors compare DNA methylation profiles of NCCs and melanocytes, as well as primary and metastatic patient tissues and identify that DNA methylation changes of NR2F2 isoform 2 influence cell state transitions and melanoma metastatic spread.

## Introduction

Growing evidence suggests that developmental differentiation programs resurface during cancer progression^1–4^. In melanoma, a highly metastatic and heterogeneous cancer, transformed melanocytic cells acquire stem cell-like features. Melanoma cells have unlimited self-renewal and multi-lineage differentiation potential, which allows them to morph into cell states with NCC-like, EMT-like, and endothelial cell features^5,6^. The ability to acquire migratory and invasive features along with a functional plasticity suggests that transformed melanocytes might de-differentiate into an NCC-like state from which they originate and develop during embryogenesis^7–9^. Indeed, transcriptomic studies have linked melanoma progression to melanocyte de-differentiation^10^ and the re-expression of markers that define NCCs^11–13^, but the mechanisms controlling this process remain largely unknown. We reasoned that some of the molecular changes that accompany the differentiation of NCCs into melanocytes could become reversed during metastatic melanoma progression. We hypothesized that the processes that control the differentiation of NCCs into melanocytes and the de-differentiation of melanoma cells into an NCC-like state could be dynamic in nature. Therefore, we focused on epigenetic changes, which can be stable but reversible at the same time. We surmised that epigenetic and transcriptional changes could orchestrate the dynamic cell state transitions that accompany the de-differentiation and adaptation of melanoma cells to the micro-environmental niches they encounter as they transition from their primary site into circulation to colonize distal metastatic sites.

Here, we compared DNA methylation profiles of NCCs and melanocytes as well as melanoma patient tissues from primary and metastatic sites. When we integrated these data, we exposed localized methylation changes in the regulatory region of NR2F2 (a.k.a. COUP-TFII), an orphan-nuclear receptor that is essential for embryonic^14^ and NCC^15^ development. We found these methylation changes control the expression of NR2F2-Iso2, a truncated NR2F2 isoform that is transcribed from an alternative upstream TSS and that lacks the DNA-binding domain. We showed *NR2F2*-Iso2 is hypomethylated in NCCs and hypermethylated in melanocytes. Conversely, *NR2F2*-Iso2 was increasingly hypomethylated from primary to metastatic melanoma. We further found that *NR2F2*-Iso2 methylation correlates inversely with *NR2F2*-Iso2 expression, being detected in NCCs but not in melanocytes and increasingly expressed from primary to metastatic melanoma tissues. Our data suggest NR2F2-Iso2 enhances melanoma metastasis by regulating the DNA-binding ability of the full-length NR2F2-Iso1, promoting the expression of EMT and NCC gene sets. Our study provides insights into how reversible changes in DNA methylation influence developmental gene expression programs and how a partial reversal of these programs restores the phenotypic plasticity that enables normal development and metastatic cancer progression.

## Results

### The *NR2F2* locus becomes methylated during NCC-to-melanocyte differentiation, and de-methylated during metastatic melanoma progression

**Fig. 1:**
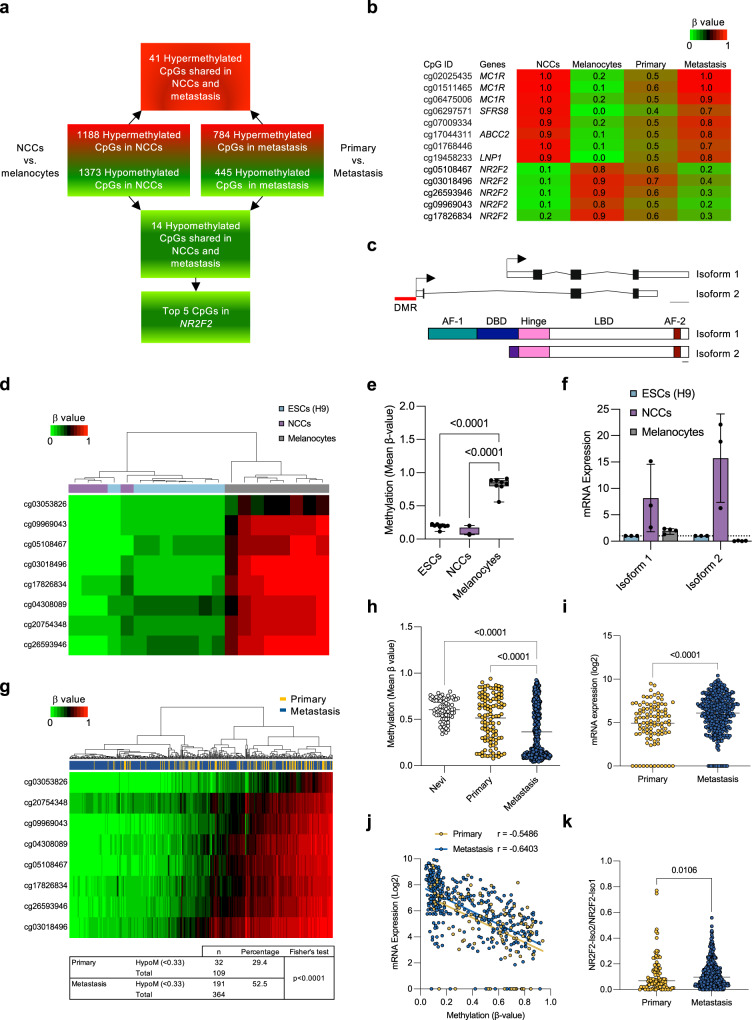
Epigenetic regulation of NR2F2 during differentiation and melanoma progression. **a** Schematic representation of a workflow to identify and integrate differentially methylated CpGs in neural crest cells (NCCs) and melanocytes along with primary and metastatic melanoma samples from TCGA. 41 CpGs are hypermethylated and 14 CpGs are hypomethylated in NCCs and metastatic melanoma. The five top-ranked hypomethylated CpGs are located in the NR2F2 locus. **b** Heatmap representation of top-ranked hypo- and hyper-methylated CpGs in NCCs vs melanocytes and metastatic vs. primary melanoma. Methylation scores (*β*-values) were determined with HumanMethylation450K arrays and they range from 0 (unmethylated, green) to 1 (completely methylated, red). **c** Schematic representation of NR2F2-Iso1 and NR2F2-Iso2 transcripts (upper panel) and proteins (lower panel). The Differentially Methylated Region (DMR, red line) in NCCs vs. melanocytes and in primary vs. metastatic samples is located at the NR2F2-Iso2 transcriptional start site (TSS; arrow). Upper and lower scale bars depict 1 kb and 10 amino acids, respectively. AF-1, 2: transactivation functional domains; DBD: DNA-binding domain; LBD ligand-binding domain. Heatmap representation of unsupervised cluster analysis of mean *β*-values (**d**) and box plots (**e** one-way ANOVA, whiskers represent min. and max.) showing NR2F2-Iso2 CpGs are unmethylated in embryonic stem (ESCs) and NCC cell lines and hypermethylated in melanocytes (*N* = 8 ESCs, *N* = 4 NCCs, *N* = 8 melanocytes). **f** Relative NR2F2-Iso1 and NR2F2-Iso2 mRNA expression in ESCs, NCCs and melanocytes by RT-qPCR (bars represent SD, *N* = 1 ESC, *N* = 3 NCCs, *N* = 4 melanocytes). **g** Heatmap representation of unsupervised cluster analysis of mean *β*-values showing NR2F2-Iso2 CpGs are more frequently hypomethylated (*β* < 0.33) in a TCGA cohort of metastatic compared to primary melanoma patient samples. 191 of 364 (52.5 %) metastatic melanoma samples were hypomethylated compared to 32 of 109 (29.4%) primary melanoma samples (Fisher’s exact test, *p* < 0.0001). **h** Scatter plots showing mean *β*-values for NR2F2-Iso2 CpGs of 73 nevi (GSE120878, ref. ^54^), 109 primary (TCGA) and 364 metastatic (TCGA) melanoma tissues (one-way ANOVA, bar represents median). **i** Scatter plots showing NR2F2-Iso2 mRNA expression in primary and metastatic melanoma samples from TCGA (two-tailed unpaired Mann–Whitney test, bar represents median). **j** Scatter plots showing inverse correlation (Spearman’s rank correlation, *p* < 0.0001) between NR2F2-Iso2 mRNA expression and CpG-methylation levels in primary and metastatic melanoma TCGA samples. CpGs depicted in heatmaps **d** and **g** are located between −300 to 63 bp relative to the NR2F2-Iso2 TSS. **k** Ratio of mRNA expression of NR2F2-Iso1/Iso2 in TCGA primary and metastasis melanoma tissues (two-tailed unpaired Mann–Whitney test, bar represents median). Source data are provided as a Source Data file.

To unbiasedly identify gene sets epigenetically regulated during the differentiation of NCCs into melanocytes and during the progression from primary to metastatic melanoma, we integrated four independent, genome-wide DNA methylation data sets that were generated with HumanMethylation450K arrays (Fig. 1a and Supplementary Fig. 1a). We first compared four human NCC explants to eight melanocyte cell cultures and we identified 1188 hypermethylated and 1373 hypomethylated CpGs in NCCs. Next, we compared the methylation profiles of 109 primary to 364 metastatic melanoma patient samples^16^ and found 784 hypermethylated and 445 hypomethylated CpGs in metastatic vs primary melanoma. We intersected these differentially methylated CpG sites and found 41 CpGs that were hypermethylated in NCCs and metastatic melanoma cells (Supplementary Fig. 1b). One of the affected genes was the pigmentation gene *MC1R* (Fig. 1b), which becomes silenced in some melanomas^17^. We also found 14 CpGs that were hypomethylated in NCCs and metastatic melanoma (Supplementary Fig. 1c). Five of the most differentially methylated CpGs (>30% change in DNA methylation) resided in *NR2F2* (Fig. 1b). *NR2F2* encodes a transcription factor that controls NCC development^15^ and vascular organization and it is essential for embryonic development in mice^18,19^. NR2F2 de-regulation has also been reported in various cancers^20–23^, where its upregulation correlates with poor clinical outcomes and metastatic progression with effects on angiogenesis, lymphangiogenesis, and tumor growth (reviewed in ref. ^24^).

### *NR2F2* demethylation restores *NR2F2*-Iso2 expression during metastatic melanoma progression

The *NR2F2* gene encodes four isoforms, Iso1 (NM_021005), Iso2 (NM_001145155), Iso3 (NM_001145156) and Iso4 (NM_001145157). *NR2F2*-Iso1 encodes the full-length protein, which contains an N-terminal activation function 1 domain (AF-1), a highly-conserved DNA-binding domain (DBD), a hinge region, and a ligand-binding domain (LBD) that contains a ligand-dependent AF-2 (Fig. 1c). NR2F2-Iso2, NR2F2-Iso3, and NR2F2-Iso4 differ from NR2F2-Iso1 in their N-terminal sequences and they lack the DNA-binding domain. The five *NR2F2* CpGs that we found to be hypomethylated in metastatic compared to primary melanomas (Fig. 1b) are located right at the *NR2F2*-Iso2 TSS (Fig. 1c).

Illumina 450K arrays (Fig. 1d, e) and bisulfite sequencing (Supplementary Fig. 2) revealed that these CpGs, along with three contiguous *NR2F2* CpGs are unmethylated in embryonic stem cells (ESCs) and NCCs, but they are fully methylated in normal melanocytes. To correlate CpG methylation with *NR2F2-*Iso2 transcription, we isolated mRNA from ESCs, NCCs and melanocytes. qRT-PCR showed an inverse correlation between *NR2F2*-Iso2 methylation and *NR2F2*-Iso2 transcription, with *NR2F2-*Iso2 mRNA found expressed in NCCs and ESCs, and silenced in melanocytes (Fig. 1f). In contrast, CpGs located within the *NR2F2-*Iso1 promoter were hypomethylated in ESCs, NCCs, and melanocytes (Supplementary Fig. 3a), and *NR2F2-*Iso1 mRNA was consistently expressed in these three cell types (Fig. 1f). Furthermore, *NR2F2*-Iso2 CpGs were more frequently hypomethylated (i.e., mean *β* value < 0.33) in metastatic (191 of 364; 52.5%) compared to primary (32 of 109; 29.4%) melanoma samples (*p* < 0.0001; Fig. 1g, h), while *NR2F2*-Iso1 CpGs were consistently hypomethylated in both, primary and metastatic samples (Supplementary Fig. 3b, c).

Furthermore, integration of global CpG methylation with mRNA expression data generated by TCGA^16^ showed that *NR2F2*-Iso2 hypomethylation correlates with the transcriptional upregulation of NR2F2-Iso2 in melanoma (*p* < 0.001; Fig. 1i, j). The ratio of NR2F2-Iso2/ NR2F2-Iso1 expression goes up from primary to metastatic melanoma (Fig. 1k).

Our data indicates that *NR2F2*-Iso2 hypomethylation is not associated with global hypomethylation (Supplementary Fig. 4a), but it does correlate with *BRAF* mutation status (*p* < 0.05; Supplementary Fig. 4b–e). In addition, *NR2F2*-Iso2 hypomethylation was found to correlate with a transcriptional signature that defines MITF-low melanoma cells, characterized by reduced expression of pigmentation and increased expression of nervous system and neuronal development associated genes^16^ (Supplementary Fig. 4f). Low MITF expression levels also correlate with increased invasion, motility, tumor forming capacity and EMT-like features^25^. Collectively, our data suggest that *NR2F2*-Iso2 becomes epigenetically repressed when NCCs differentiate into melanocytes and it becomes aberrantly re-expressed during metastatic melanoma progression.

**Fig. 2:**
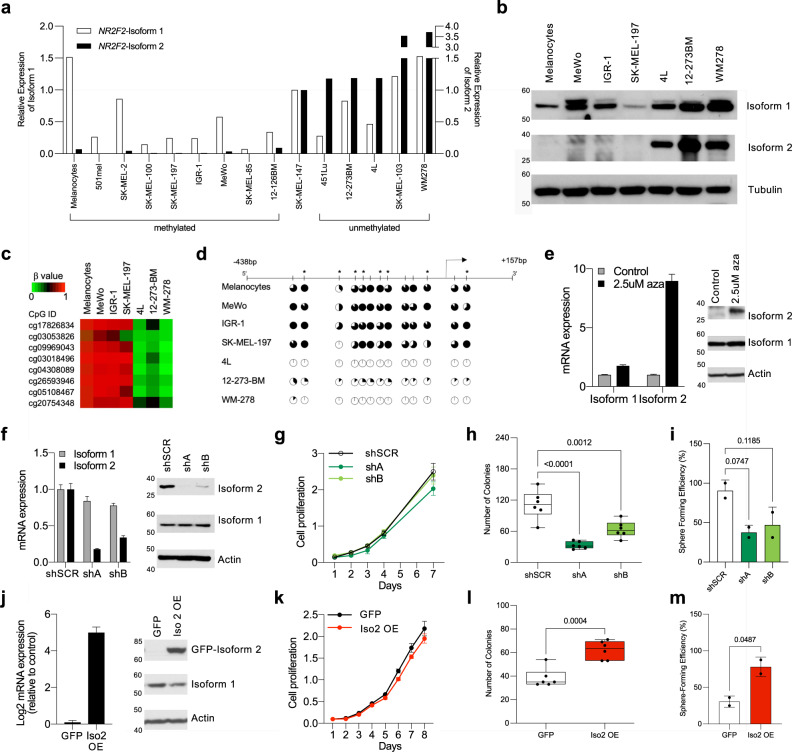
NR2F2-Iso2 promotes anchorage-independent growth and melanoma sphere formation in vitro. **a** qRT-PCR showing relative NR2F2-Iso1 and NR2F2-Iso2 transcript levels normalized to SK-MEL-147, where NR2F2-Iso2 is partly methylated (M) and unmethylated (U). Methylation status was determined with Illumina 450 K arrays and/or methylation specific PCR. **b** Western blots of melanoma cell lines probed with Isoform-specific NR2F2 antibodies. **c** Heatmap illustrating CpG-methylation status of NR2F2-Iso2 based on Illumina 450 K array data. **d** Pie charts showing % CpG methylation of indicated sites at the NR2F2 locus as determined by bisulfite sequencing. Asterisks denote CpGs interrogated in 450 K arrays. **e** qRT-PCR (left) and western blotting (right) show that treatment of MeWo cells with the demethylating agent 5-aza-2′-deoxycytidine (2.5 μM aza, 72 h) permits NR2F2-Iso2 expression (bars represent SD). One experiment of 2 biological replicates is shown; further validated in other cell lines in Supplementary Fig. 7. **f** qRT-PCR and western blotting validate isoform-specific NR2F2-Iso2 depletion in NR2F2-Iso2 expressing 4L cells with shNR2F2-Iso2 (shA, shB) compared to shSCR (control) (bars represent SD). Actin served as loading control. Representative result from one of multiple repeats is shown (>3). **g** Growth rates of shSCR and shNR2F2-Iso2 expressing 4L cells showing no significant differences in 2D cultures (two-way ANOVA, bars represent SD). **h** Bar graph showing significant difference in colony forming potential in soft agar between shSCR and shNR2F2-Iso2 expressing 4 L cells 21 days after seeding (one-way ANOVA, bars represent min. and max.). **i** Bar graphs showing changes in sphere forming potential between shSCR and shNR2F2-Iso2 expressing 4L cells (one-way ANOVA, bar represents SD). **j** qRT-PCR and western blotting validate ectopic NR2F2-Iso2 and endogenous NR2F2-Iso1 expression in MeWo cells (bar represents SD). Actin served as loading control. Representative result from one of multiple repeats is shown (>3). **k** Growth rates of GFP control and NR2F2-Iso2 overexpressing (Iso2 OE) MeWo cells showing no significant differences in 2D cultures (two-way ANOVA, bar represents SD). **l** Box plots showing significant difference in colony forming potential in soft agar between GFP control and NR2F2-Iso2 overexpressing MeWo cells 28 days after seeding (two-tailed unpaired *T*-test, bar represents min. and max.). **m** Bar graphs showing changes in sphere forming potential between GFP control and NR2F2-Iso2 overexpressing MeWo cells (two-tailed unpaired *T*-test, bar represents SD). (**h**, **i**, **l**, **m**: One of three independent experiments is represented). Source data are provided as a Source Data file.

To further interrogate the inverse correlation between *NR2F2*-Iso2 methylation and expression, we analyzed a panel of melanoma cell lines (Fig. 2a, b; Supplementary Fig. 5) and patient-derived short-term cultures^26^ (STCs, Fig. 2a, b; Supplementary Fig. 6). Both qRT-PCR (Fig. 2a) and western blotting (Fig. 2b) detected NR2F2-Iso2 expression in cultures where *NR2F2*-Iso2 CpGs were hypomethylated as measured by 450 K Illumina arrays (Fig. 2c; Supplementary Fig. 5a, Supplementary Fig. 6a) and bisulfite sequencing (Fig. 2d; Supplementary Fig. 5b, Supplementary Fig. 6b). To determine whether CpG methylation prevents *NR2F2*-Iso2 expression, we treated two melanoma cell lines and two STCs with the DNA demethylating agent 5-aza-2′-deoxycytidine for 72 h. qRT-PCR and western blotting showed that 5-aza treatment increased NR2F2-Iso2 expression (Fig. 2e, Supplementary Fig. 7), supporting the idea that CpG demethylation is required for the re-expression of NR2F2-Iso2 in melanoma cells.

### NR2F2-Iso2 promotes melanoma cell survival and anchorage-independent growth

To functionally test the contribution of *NR2F2*-Iso2 to metastatic melanoma progression we performed loss-of-function (LOF) and gain-of-function (GOF) experiments. Building on our *NR2F2-Iso2* methylation and expression data (Fig. 2a–d**;** Supplementary Figs. 5, 6), we selected cell lines in which *NR2F2*-Iso2 is methylated (i.e., MeWo, IGR-1, SK-MEL-197) or unmethylated (i.e., WM239A-derived 113/6-4L cells^27^, hereafter 4L; WM278, and patient-derived STC 12-273BM). We stably transduced these melanoma cells with lentiviral vectors to constitutively express fluorescent or luciferase reporters to monitor the cells in vitro and in vivo, respectively. Next, we stably transduced 4L, 12-273BM, and WM278 cells to express scrambled (shSCR) control or *NR2F2*-Iso2 (shA, shB) shRNAs. qRT-PCR and western blotting showed NR2F2-Iso2 was selectively depleted by shRNA (Fig. 2f, Supplementary Fig. 8a, b) and this depletion had no effect on the cells’ growth rate in two-dimensional (2D) cultures (Fig. 2g; Supplementary Fig. 8c), but it significantly suppressed the cells’ ability to form colonies in soft agar and spheres from single cell suspensions in low attachment plates (Fig. 2h, i).

We also transduced MeWo, IGR-1, and SK-MEL-197 cells, in which *NR2F2*-Iso2 is hypermethylated and silenced, with an ectopic *NR2F2*-Iso2 expression vector. qRT-PCR and western blotting confirmed ectopic NR2F2-Iso2 expression in MeWo cells (Fig. 2j), which had no effect on the cells’ growth rate in 2D cultures (Fig. 2k), but significantly increased their ability to form colonies in soft agar (Fig. 2l) and spheres from single cells in low attachment plates (Fig. 2m). Similar results were obtained in IGR-1, and SK-MEL-197 cells engineered to overexpress NR2F2-Iso2 (Supplementary Fig. 9). Together, these data suggest that NR2F2-Iso2 expression increases anchorage-independent growth and sphere formation from single cells, two features widely considered reflective of enhanced survival under challenging conditions, which can benefit melanoma cells when they colonize distant metastatic sites.

### NR2F2-Iso2 enhances melanoma metastasis

**Fig. 3:**
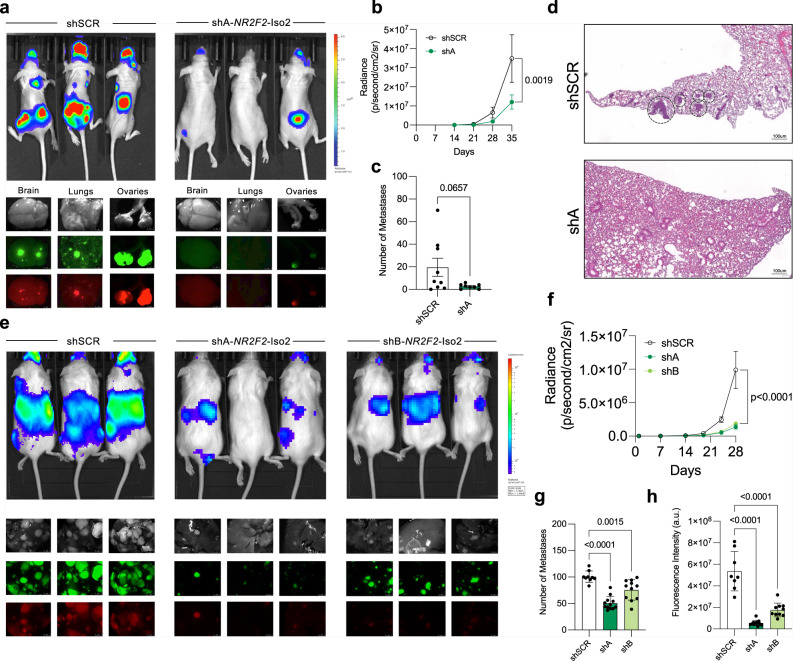
NR2F2 isoform 2 promotes melanoma metastasis. **a**–**d** 4L (*N* = 9 shSCR, *N* = 8 shA athymic/nude mice) and **e**–**h** 12-273BM (* N*= 9 shSCR, *N* = 12 shA, *N* = 11 shB NSG mice) cells  labeled with lentiviruses that constitutively express luciferase and mCherry, and transduced with lentiviruses expressing shSCR or shNR2F2-Iso2 (shA, shB) along with green fluorescent protein (GFP), were instilled into mouse hearts by ultrasound-guided injection. **a**, **e** Bioluminescence and fluorescence images of mice and their corresponding organs (brains, lungs and ovaries in **a**; livers in **e**) ex vivo at the endpoint of one representative experiment. **b**, **f** In Vivo Imaging System (IVIS) measurements showing significant differences in radiance levels between groups during tumor progression (two-way ANOVA, bars represent SEM). **c** Bar graphs showing that the average number of lung metastases per tissue section is significantly reduced in mice injected with shNR2F2-Iso2 compared to those injected with shSCR expressing 4L cells (two-tailed unpaired T-test, bars represent SEM). **d** Representative H&E-stained tissue sections. Circles identify metastases. Scale bar = 100 μm. Bar graphs showing **g** average number of metastases per liver section and **h** GFP intensity of livers indicate significantly reduced metastatic burden in mice injected with shNR2F2-Iso2 compared to those instilled with shSCR expressing 12-273 BM cells (two-way ANOVA, bars represent SD). Error bars represent standard error of the mean. Source data are provided as a Source Data file.

To functionally test whether NR2F2-Iso2 expression affects melanoma growth in xenograft models, we transplanted shSCR or shNR2F2-Iso2 expressing 4L cells subcutaneously into NSG mice to measure differences in tumor growth over time. Consistent with our proliferation data in 2D cultures, we found no significant difference in the growth rate of subcutaneous shSCR or shNR2F2-Iso2 expressing 4L tumors (Supplementary Fig. 8d). However, when we instilled these cells by ultrasound-guided intracardiac injection into mice, we observed a significantly decreased (*p* = 0.002) metastatic potential in shNR2F2-Iso2 compared to shSCR expressing 4L cells (Fig. 3a–d). A similar effect was observed in patient-derived 12-273BM (Fig. 3e–h) and WM278 cells (Supplementary Fig. 10) after transduction with two independent *NR2F2*-Iso2 shRNAs.

Conversely, ectopic NR2F2-Iso2 expression enhanced the metastatic potential of MeWo cells compared to controls in our intracardiac injection model (Supplementary Fig. 11), or in the subcutaneous transplantation model after we surgically resected tumors that grew at the transplantation site (Supplementary Fig. 12). Collectively, our correlative studies with clinical specimens and functional studies in xenograft models identified NR2F2-Iso2 as a metastasis driver in melanoma.

### NR2F2-Iso2 controls a pro-metastatic transcriptional program

**Fig. 4:**
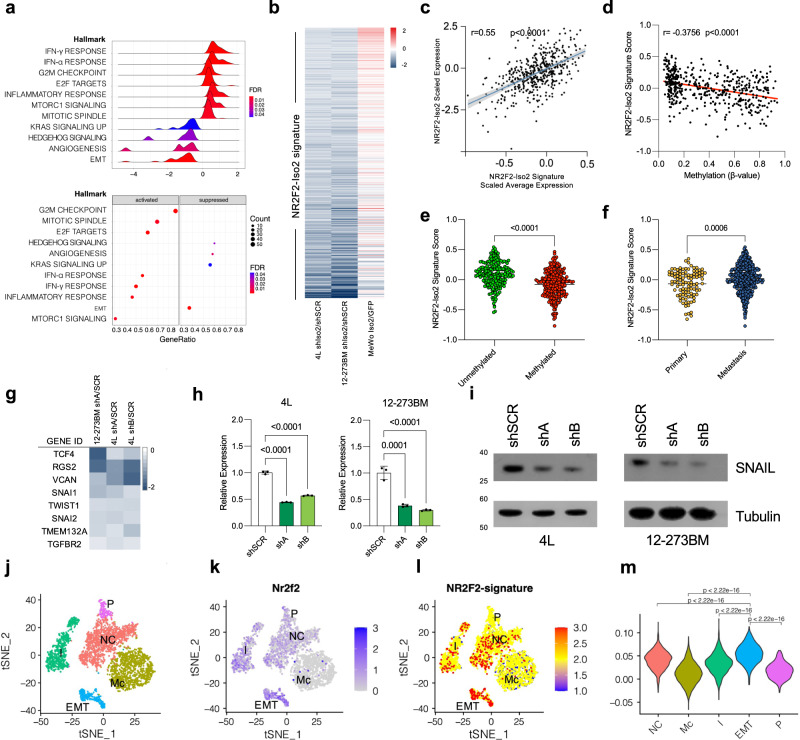
NR2F2-Iso2 controls an expression signature enriched in metastasis promoting gene sets. **a** Ridge and Bubble plots showing transcriptomic changes after NR2F2-Iso2 loss in 4L cells are significantly associated with reduced angiogenesis and EMT gene sets. p.adj indicates FDR. **b** Heatmap showing Log2 fold changes of 500 NR2F2-Iso2 signature genes that were significantly down-regulated in 4L and 12-273BM cells upon NR2F2-Iso2 knockdown. Most of these genes are up-regulated in MeWo cells upon ectopic NR2F2-Iso2 expression. Scatter plots and Spearman tests (*p* < 0.001) correlate average NR2F2-Iso2 signature gene expression directly with NR2F2-Iso2 expression (**c**) and indirectly with NR2F2-Iso2 CpG methylation (**d**) in human melanoma samples from TCGA. Scatter plots showing the NR2F2-Iso2 signature gene score is significantly higher in unmethylated compared to methylated (**e**) and metastatic compared to primary (**f**) melanoma samples from TCGA (two-tailed Mann–Whitney test, bar represents median). **g** Heatmap showing Log2 fold-change expression of selected EMT genes between shNR2F2-Iso2 and shSCR expressing 4L or 12-273BM cells. **h** qRT-PCR and **i** western blotting show significantly reduced SNAI1 expression in shNR2F2-Iso2 compared to shSCR expressing 4L or 12-273BM cells. (*N* = 3, two-way ANOVA, data shown from one representative experiment; bars represent SD; tubulin served as western blot loading control). **j**–**m** tSNE and Violin plots showing scRNA-seq data from the Tyr-CreER; BRAF^CA/+^/ Pten^FL/FL^;R26-LSL-tdTomato mouse model [ref. ^31^.]. **j** Dimensionality plot identifies neural crest-like (NC), melanocytic (Mc), intermediate (I), EMT-like, and proliferative (P) melanoma cells. Feature plots show enrichment of (**k**) Nr2f2 expression and (**l**) NR2F2-Iso2 signature score in melanoma cell states. **m** Violin plots show Nr2f2-Iso2 signature gene enrichment in EMT-like cells (two-way unpaired Mann-Whitney test). Source data are provided as a Source Data file.

To determine how NR2F2-Iso2 enhances metastatic melanoma progression, we first profiled our LOF models with RNA-seq, identified genes that were differentially expressed between shNR2F2-Iso2 and shSCR expressing cells (*p* < 0.05) and ranked them from their highest to lowest fold-change expression for gene set enrichment analyses (GSEA) with the Hallmark reference gene sets. These analyses suggested that *NR2F2*-Iso2 loss inhibits angiogenesis and EMT, which have previously been linked to metastatic melanoma progression^28^ (Fig. 4a). To probe deeper into NR2F2-Iso2 regulated genes in melanoma, we identified NR2F2-Iso2 signature genes that were consistently down-regulated in shNR2F2-Iso2 compared to shSCR in 4L and 12-273BM cells (Fig. 4b; Supplementary Data 1). The majority of these genes were upregulated upon ectopic NR2F2-Iso2 expression in MeWo cells (Fig. 4b). The scaled average expression of these NR2F2-Iso2 signature genes correlated directly (*r* = 0.55, *p* < 0.001) with the scaled *NR2F2*-Iso2 expression (Fig. 4c) and inversely (*r* = −0.38, *p* < 0.001) with *NR2F2*-Iso2 methylation status (Fig. 4d, e) across melanoma patient samples from TCGA. The expression of these signature genes was also significantly increased (*p* < 0.01) in metastatic compared to primary melanoma patient samples from TCGA (Fig. 4f). Within this signature gene set we noticed *SNAI1* (SNAIL), *SNAI2* (SLUG), *VCAN*, and *TWIST1 (*Fig. 4g), which had previously been linked to EMT. RT-qPCR and western blotting further confirmed the downregulation of SNAIL in shNR2F2-Iso2 compared to shSCR expressing 4L or 12-273BM cells *(*Fig. 4h, i). Collectively, these data suggest that epigenetic *NR2F2*-Iso2 re-activation supports the expression of EMT genes in human melanoma.

Melanoma cells are remarkably heterogeneous in individual patients^29,30^ and genetically-engineered melanoma models^31^, where single cell RNA-seq (scRNA-seq) revealed melanoma cell states with melanocytic (Mc), NC-, and EMT-like features (Fig. 4j). To test whether NR2F2-Iso2 is active in a particular cell state, we visualized the expression of *Nr2f2* and NR2F2-Iso2 signature genes (Fig. 4b; Supplementary Data 1) on the recently reported Tyr-Cre^ER^; *BRAF*^*CA/+*^*/Pten*^*FL/FL*^;R26-LSL-tdTomato scRNA-seq data set^31^. We found that *Nr2f2* and NR2F2-Iso2 signature genes are significantly enriched in NC- and EMT-like melanoma cell states (Fig. 4k–m). These results support the idea that NR2F2-Iso2 drives a transcriptional program that elicits metastatic features in a subset of melanoma cells.

### NR2F2-Iso2 modulates NR2F2-Iso1 chromatin binding and target gene expression

**Fig. 5:**
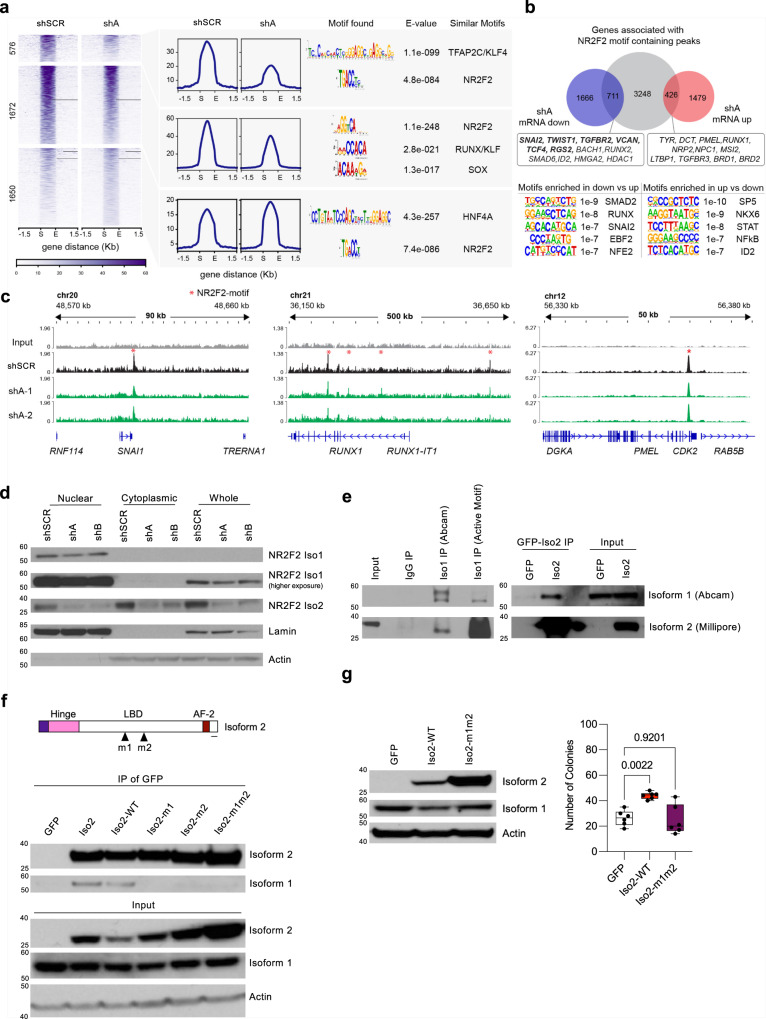
NR2F2-Iso2 modulates the transactivation capacity of full-length NR2F2-Iso1. **a** Heatmaps and histograms of NR2F2-Iso1 ChIP-seq peaks that significantly decrease (*p* < 0.05), modestly decrease (*p* > 0.05) or increase (*p* < 0.05) upon NR2F2-Iso2 silencing. MEME-ChIP discovers centrally distributed NR2F2-like motifs at peak summits in each cluster along with other transcription factor motifs that are enriched with NR2F2 in specific clusters. **b** Venn diagram intersecting 4385 potential NR2F2 target genes (annotated with GREAT—Genomic Regions Enrichment of Annotations Tool—using standard parameters) with 1905 up- or 2377 downregulated transcripts in shNR2F2-Iso2 compared to shSCR melanoma cells suggest NR2F2-Iso2 loss inhibits the expression of 711 and activates the expression of 426 direct NR2F2-Iso1 targets. HOMER identifies transcription factor motifs that are significantly enriched at NR2F2-bound sites that result in decreased or increased target gene transcription after NR2F2-Iso2 loss. **c** Examples of NR2F2-Iso1 ChIP-seq tracks in 4L cells transduced with scrambled or shA (2 experimental replicates shown) showing NR2F2-Iso1 binding to the regulatory regions of genes modulated by NR2F2-Iso2 SNAI1, RUNX1, and PMEL. **d** Cellular fractionation studies identify NR2F2-Iso2 in the nuclear and cytoplasmic fractions. NR2F2-Iso1 was exclusively detected in the nuclear fraction of 4L cells. **e** Two NR2F2-Iso1 antibodies (Abcam, Active Motif) co-immunoprecipitate NR2F2-Iso2 along with NR2F2-Iso1 from 4L cell lysates. Likewise, GFP Trap (ChromoTek) immuno-precipitates NR2F2-Iso1 along with ectopically expressed GFP-NR2F2-Iso2 from MeWo cell lysates. Immunoprecipitated NR2F2-Iso1 was detected by western blot with NR2F2-Iso1 (antibody Abcam) and immunoprecipitated NR2F2-Iso2 was detected with NR2F2-Iso2 specific antibody (Millipore). **f** MeWo cells were transduced with lentiviral vectors expressing GFP (pLenti-C-mGFP), GFP-NR2F2-Iso2 (Iso2, Iso2-wt) or GFP-NR2F2-Iso2 mutants where Leucine 231 (Iso2-m1), Leucine 232 (Iso2-m2) or both (Iso2-m1m2) were mutated to Alanine. GFP antibodies immunoprecipitated NR2F2-Iso1 with wild-type GFP-NR2F2-Iso2 but not with their mutants after their expression in MeWo cells. **g** Box plots (right) showing that ectopic expression of GFP-NR2F2-Iso2 (Iso2-WT), but not GFP or GFP-NR2F2-Iso2 where Leucine 231 and Leucine 232 were mutated to Alanine (Iso2-m1m2), enhances the colony forming potential of MeWo cells significantly (two-way ANOVA, one of three independent experiments is represented, bars represent min. and max.). NR2F2-isoform specific expression in GFP, Iso2-WT and Iso2m1m2 was assessed by western blot (left). Experiments in **f**, **g** have been done twice. Source data are provided as a Source Data file.

Although NR2F2 has been shown to promote tumorigenesis and metastasis in other cancers^20^, the function of NR2F2 isoforms remains unknown in these contexts. Because NR2F2-Iso2 doesn’t contain the DNA-binding domain but it retains the NR2F2 dimerization domain, we hypothesized that it could bind NR2F2-Iso1 and modulate its activity. Two prior studies attempted to address this potential interplay. One study suggested NR2F2-Iso2 enhances NR2F2-Iso1 transactivation activity on the *EGR1* locus in human ESCs^32^. In contrast, the other study proposed NR2F2-Iso2 has dominant-negative functions and it inhibits NR2F2-Iso1 at the *Cyp7a1* locus in hepatocellular carcinoma^33^. These differences could be explained by cell type or target gene specific NR2F2-Iso2 effects. Therefore, we took an unbiased approach and defined how NR2F2-Iso2 loss affects the NR2F2-Iso1 chromatin binding profile. We performed chromatin immunoprecipitation of NR2F2-Iso1 followed by sequencing (ChIPseq) on 4L-SCR and 4L-shNR2F2-Iso2 cells. Next, we identified ChIP-seq signals compared to input controls with MACS (*p* < 0.05, >5-fold enrichment) for each condition. De novo motif analyses with MEME-ChIP revealed a highly significant enrichment of NR2F2-like motifs at peak centers, which validated the specificity of our ChIPseq data (Fig. 5a). Amongst these motif-containing peaks in our 4L-shSCR and 4L-shNR2F2-Iso2 ChIPseq profiles, we identified 576 NR2F2-Iso1 peaks that weakened significantly along with 1672 peaks that decrease modestly after NR2F2-Iso2 depletion. However, we also detected 1650 peaks that increased in shNR2F2-Iso2 compared to shSCR expressing 4L cells (Fig. 5a). MEME-ChIP analysis with peaks of each cluster identified a highly significant enrichment of NR2F2-like motifs at their peak summits, along with transcription factor motifs that distinguished the clusters (Fig. 5a, right panel). These data suggest that NR2F2-Iso2 can both enhance or reduce NR2F2-Iso1 binding in a context-dependent manner and we speculate that these differences could be due to gained or lost interactions with other transcription factors or chromatin modifying enzymes.

### NR2F2-Iso2 modifies NR2F2-Iso1 transcriptional regulation of metastasis and differentiation genes

Although NR2F2 was initially perceived as a transcriptional repressor^34^, it should be considered a cell type- and context-dependent transcriptional suppressor or activator^14^. To unbiasedly address this notion in melanoma, we began to identify gene sets that were directly regulated by NR2F2-Iso1 in an NR2F2-Iso2-dependent manner. We focused on ChIP-seq peaks with NR2F2-like motifs and predicted 4385 potential NR2F2-Iso1 target genes using GREAT^35^. Of these potential target genes, we identified 711 of 2377 down-regulated, and 426 of 1905 up-regulated genes that changed significantly (*p* < 0.05) in their expression upon *NR2F2*-Iso2 depletion (Fig. 5b and Supplementary Data 2). Interestingly, 197 of the 711 down-regulated NR2F2-Iso1 target genes were part of the ‘NR2F2-Iso2 signature’. Amongst these NR2F2-bound and NR2F2-Iso2 function dependent transcripts, we found known regulators of EMT and pro-metastatic genes, including *FUT8*^36^, *HMGA2, GAS7*, ***MEF2C***^37^, ***HEY2***, *ID2*, ***TGFBR2***, ***ITGA4****, PDGFRA, NRP1, PREX1, SOX2*, ***VCAN***, ***TWIST1***, or ***SNAI2*** (Supplementary Data 2; bold font denotes genes in the ‘NR2F2-Iso2 signature’ we defined in Fig. 4b and Supplementary Data 1). Conversely, genes that were inhibited by NR2F2-Iso2 function include regulators of melanocyte differentiation and pigmentation such as *PMEL, TYR*, or *DCT*. Next, we focused on these potential direct NR2F2-Iso1 targets that are NR2F2-Iso2 dependent. Using HOMER (Hypergeometric Optimization of Motif EnRichment)^38^, we uncovered differentially enriched transcription factor motifs within NR2F2-bound regulatory elements in transcripts up- or down-regulated upon NR2F2-Iso2 silencing (Fig. 5b, low panels). Together, these data suggest that NR2F2-Iso2 expression modifies NR2F2-Iso1 activity and provides evidence that NR2F2 functions as a transcriptional enhancer or repressor in a context-dependent manner.

Our analyses exposed a pro-metastatic program in melanoma where aberrantly expressed NR2F2-Iso2 regulates the transactivating capacity of NR2F2-Iso1 over differentiation (e.g., *PMEL*) and metastasis (e.g., *SNAI1*) - regulating gene sets (Fig. 5c). In support of this model, we identified both NR2F2-Iso1 and NR2F2-Iso2 in the nuclear fraction, although NR2F2-Iso2 is also detected in the cytoplasmic fraction (Fig. 5d). NR2F2-Iso2 is able to translocate to the nucleus congruent with a nuclear localization signal (NLS) in the Hinge region that both isoforms share, although it doesn’t contain the stronger NLS in the DNA-binding region (Fig. 1c). To determine whether NR2F2-Iso2 interacts with NR2F2-Iso1, we co-immunoprecipitated endogenously expressed NR2F2-Iso2 with NR2F2-Iso1 in 4L cells, and ectopically expressed GFP-NR2F2-Iso2 with endogenously expressed NR2F2-Iso1 in MeWo cells (Fig. 5e). Next, we mutated Leu231 and Leu232 in the dimerization domain into alanines^39^ which inhibited the ability of NR2F2-Iso2 to interact with NR2F2-Iso1 (Fig. 5f), and it was no longer able to enhance anchorage-independent growth (Fig. 5g). This finding suggests that the pro-metastatic activity of NR2F2-Iso2 is dependent on its ability to dimerize with NR2F2-Iso1 and the formation of NR2F2-Iso1/NR2F2-Iso2 heterodimers during melanoma progression may alter interactions with other nuclear receptors, transcription factors or chromatin modifiers, resulting in the expression of metastasis promoting genes in melanoma cells (Supplementary Fig. 13).

### Shifting the ratio of NR2F2 isoforms changes melanoma metastatic potential

**Fig. 6:**
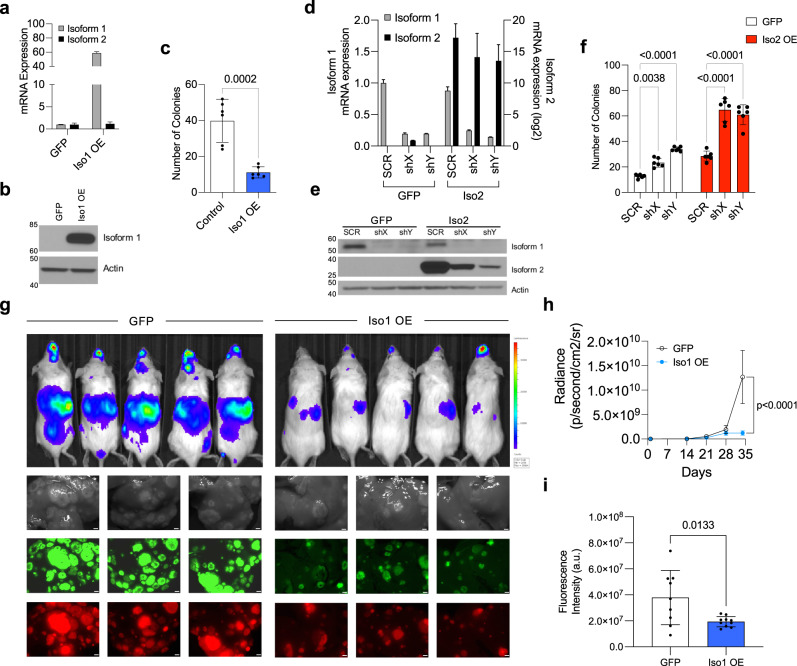
NR2F2-Iso1 reduces colony formation ability and metastatic potential. **a** qRT-PCR and **b** western blotting confirms ectopic NR2F2-Iso1 expression in MeWo cells (bars represent SD). **c** Bar graph showing significantly reduced colony forming ability of MeWo cells that ectopically express NR2F2-Iso1 28 days after seeding (two-tailed unpaired T test, bars represent SD). **d** qRT-PCR data and **e** western blotting showing differences in NR2F2-Iso1 expression between shSCR and shNR2F2-Iso1 (shX, shY) MeWo cells that ectopically express GFP or GFP-NR2F2-Iso2 (bars represent SD). **f** Bar graphs showing relative colony forming efficiencies of these cells 21 days after seeding (two-way ANOVA, bars represent SD) (*n* = 6 experimental replicates). Experiments in **a**–**f** have been done al least twice; **c** and **f** three times. (**g**–**i**) 4L cells (*n* = 9 GFP, 10 Iso1 OE NSG mice) labeled with lentiviruses that constitutively express luciferase and red fluorescence protein (RFP) and transduced with lentiviruses expressing GFP or GFP-NR2F2-Iso1, were instilled into NSG mouse hearts. **g** Bioluminescence and fluorescence images of mice and their corresponding metastases containing livers ex vivo at the endpoint of one representative experiment. **h** In Vivo Imaging System (IVIS) measurements showing significant reduction in radiance levels in mice injected with GFP-NR2F2-Iso1 cells compared to those harboring GFP expressing cohorts during tumor progression (two-way ANOVA, bars represent SEM). **i** Bar graphs showing average GFP intensity in livers of mice injected with GFP or GFP-NR2F2-Iso1 expressing 4L cells (two-tailed unpaired T-test, bars represent SD). Scale bars = 1 cm on macroscopic images of livers. Source data are provided as a Source Data file.

To test whether NR2F2-Iso2/NR2F2-Iso1 interactions promote anchorage-independent growth and metastatic progression, we transduced MeWo cells (that do not express NR2F2-Iso2) with GFP or NR2F2-Iso1. Ectopic NR2F2-Iso1 expression (Fig. 6a, b) significantly reduced colony forming potential (Fig. 6c). Next, we expressed shSCR or shNR2F2-Iso1 (shX, shY) in MeWo cells that either expressed GFP or NR2F2-Iso2. We confirmed changes in NR2F2-Iso1 and NR2F2-Iso2 expression by qRT-PCR (Fig. 6d) and western blotting (Fig. 6e) and measured the cells colony forming potential (Fig. 6f). We found that ectopic NR2F2-Iso2 expression increases the colony forming potential of MeWo cells and NR2F2-Iso1 depletion enhanced this potential even further. These data suggest that the NR2F2-Iso2 and NR2F2-Iso1 expression ratio influences the colony forming potential of melanoma cells.

To test this hypothesis in a different cell type, we ectopically expressed GFP or GFP and NR2F2-Iso1 in 4L cells (which express NR2F2-Iso2) before we instilled these cells into the left ventricle of the heart to measure differences in their metastatic potential. We observed significantly reduced bioluminescence and fluorescence levels in mice injected with NR2F2-Iso1- compared to GFP- expressing 4L cells (Fig. 6g–i). Collectively, our studies suggest that an increasing ratio of NR2F2-Iso2 over NR2F2-Iso1 increases the colony forming and metastatic potential of melanoma cells, and that these features are dynamically regulated dependent on the methylation of CpGs at the NR2F2-Iso2 TSS.

## Discussion

Our study unearthed a mechanism by which epigenetic changes in DNA methylation inhibit NR2F2-Iso2 expression in melanocytes and enable its re-expression in human metastatic melanoma. Seminal studies in a zebrafish model suggested that melanocytic cells de-differentiate as they acquire epigenetic changes along with NCC-like features during melanoma formation^13,40^. Although NCC-like features have been reported in human melanoma, it was still unknown whether and how changes in DNA methylation affect human melanoma development and metastatic melanoma progression. We reasoned that CpG-methylation changes that occur during NCC-to-melanocyte differentiation might become reversed when primary melanoma cells become metastatic. Therefore, we compared global DNA methylation profiles of human NCC and melanocyte cultures as well as primary and metastatic human melanomas. We uncovered 1373 hypomethylated and 1188 hypermethylated CpGs when we compared NCCs to melanocytes. In addition, we found 445 hypomethylated and 784 hypermethylated CpGs when we compared human metastatic to primary melanomas. Surprisingly, only 14 CpGs were consistently hypomethylated in NCCs and metastatic melanomas. Five of these CpGs were located at the *NR2F2*-Iso2 TSS, where their methylation prevents *NR2F2*-Iso2 transcription. These data suggest that metastatic melanoma progression is not simply a reversal of the NCC-to-melanocyte differentiation program but metastatic melanoma cells may rather acquire a distinct set of molecular features. This idea is consistent with squamous cell carcinoma models where epigenetic and transcriptional changes result in a “lineage-infidelity” that promotes cancer development^41,42^ and metastatic progression^43^. Alternatively, technical issues inherent to the approach (e.g., use of samples that are difficult to compare: human NCCs, neonate melanocytes, and tissue specimen from different donors) may have impaired our ability to observe a more general re-activation of neural crest epigenetic programs during metastatic progression. Although our studies do not support a general turnaround of the melanocyte-to-NCC CpG-methylation profile during metastatic melanoma progression, they suggest metastatic progression depends on the reversal of NR2F2-Iso2 repression.

Although transcriptional changes during cancer progression are intensively studied, isoform-specific changes are rarely considered. However, a few examples of isoform switching during metastatic cancer progression have been reported. For example, a cryptic transcript of the Rab GTPase activating protein TBC1D16 was noticed in melanoma^44^, and a switch between long and short Tks5 isoforms was reported in lung adenocarcinoma^45^. However, their regulation and function are not as sophisticated as the regulatory function of NR2F2, where NR2F2-Iso1 appears to function as a putative metastasis suppressor and this activity can be inhibited by the expression of NR2F2-Iso2, which is transcribed from an alternative TSS and regulated epigenetically by focal changes in DNA methylation (Supplementary Fig. 13). This elegant mechanism appears to auto-regulate NR2F2 activity and may support reversible phenotypic changes and plasticity in melanoma, in addition to irreversible genetic alterations that drive tumor initiation and progression. Our study suggests that isoform-specific analyses of RNA sequencing data, along with complementary DNA methylation profiles and isoform-specific functional genomics data will be necessary to better understand how phenotypic changes are regulated in melanoma and other cancer types. Our results also call for a more detailed analysis of NR2F2-isoform-specific functions in NCC^15^, reproductive tract development^46^, and other cancer types^20,21,23^.

NR2F2-Iso2 is a truncated isoform of NR2F2-Iso1. It is expressed from an alternative TSS and it lacks the N-terminal DNA-binding domain that is unique to NR2F2-Iso1. Our biochemical studies showed that NR2F2-Iso2 interacts physically with the C-terminal NR2F2-Iso1 AF-2 domain. Previous studies suggested that NR2F2-Iso1/Iso2 interactions promote the expression of the *EGR1* locus in human ESCs^32^. In contrast, this interaction was found to inhibit the expression of *Cyp7a1* in hepatocellular carcinoma^33^. Taking a global, unbiased approach, our NR2F2-Iso1 ChIP-seq and RNA-seq studies in control and shNR2F2-Iso2 expressing melanoma cells suggest NR2F2-Iso2 affects NR2F2-Iso1 target gene expression in a context-dependent manner. Although we found a few sites where NR2F2-Iso1 chromatin interaction was dependent on NR2F2-Iso2 expression, the majority of NR2F2-Iso1 bound sites was unaffected by NR2F2-Iso2 depletion. Furthermore, we found a similar subset of potential NR2F2-Iso1 target genes increased or decreased upon NR2F2-Iso2 depletion suggesting that NR2F2-Iso2 does not simply function as a transcriptional enhancer or repressor. Instead, we found SMAD2, RUNX and SNAI2 motif enrichment at genes that declined upon NR2F2-Iso2 loss and NKX6, STAT and NFκB motif enrichment at genes whose expression increased upon NR2F2-Iso2 loss. These data suggest NR2F2-Iso1 interacts with NR2F2-Iso2 and other transcription factors to elicit context-dependent transcriptional changes. This idea is also supported by a recent study which analyzed NR2F2-ChIP-seq and RNA-seq data in control and NR2F2-inhibitor-treated prostate cancer samples^47^. It is interesting to note that the pharmacological NR2F2 inhibitor also binds to the AF-2 domain and it likely inhibits the interaction of NR2F2-Iso1 with transcriptional partners and target gene expression, without affecting its chromatin binding pattern.

Although a significant fraction of NR2F2-Iso2 dependent transcriptional changes can be directly linked to altered NR2F2-Iso1 activity, it is formally possible that NR2F2-Iso2 also affects NR2F2-Iso1 independent functions. This idea is primarily rooted in NR2F2-Iso1 knockdown studies with and without NR2F2-Iso2 ectopic expression. These studies revealed that increased NR2F2-Iso2 expression promotes colony formation and metastatic dissemination and NR2F2-Iso1 knockdown escalates this effect. In contrast, ectopic NR2F2-Iso1 expression inhibits colony formation and metastatic dissemination. Although our data favor a model whereby NR2F2-Iso2 interacts with NR2F2-Iso1 to convert its metastasis inhibitory into metastasis promoting functions, we cannot rule out that NR2F2-Iso2 promotes metastatic dissemination by other means.

Future studies will need to determine how NR2F2-Iso2 expression affects the interaction of NR2F2-Iso1 with other transcription factors and how these interactions fine tune the metastatic progression program. However, we already found NR2F2-Iso2 enhances the expression of EMT and angiogenesis regulatory gene sets in human metastatic melanoma. EMT and angiogenesis have long been linked to metastatic dissemination in multiple cancers and scRNA-seq revealed NCC-like and EMT-like cell states in primary melanoma mouse models^31^. Retrospective and prospective studies will be required to determine whether NR2F2-Iso2 expression in primary melanomas could function as a prognostic marker for their metastatic dissemination and whether patients with NR2F2-Iso2 re-expression would benefit from treatment with pharmacological NR2F2 inhibitors or other adjuvant treatment approaches.

## Methods

### Cell lines and culture

The H9 embryonic stem cell line was purchased from WiCell Institute and maintained in co-culture with MitC-treated primary mouse embryonic fibroblasts (MEFs) in the presence of FGF2 (6 ng/ml; R&D Systems), under conditions described by the supplier. Neural crest-derived primary cell lines (NCCs) were isolated in accordance with institutional authorities’ guidelines and French legal regulations (Bioethics law 2004-800 and Protocol PFS14-011), as described^48,49^. NCC1 (90003), NCC2 (SZ08) and NCC3 (SZ15) were derived from 7th post-conceptional week (PCW) dorsal root ganglia (DRG), while NCC4 (SZ112) was derived from migratory Schwann cell precursors explanted from an 11th PCW brachial plexus. All NCCs were grown in collagen I (BD bioscience)-coated plates using the following medium: Dulbecco’s Modified Eagle Medium/Nutrient Mixture F-12 with GlutaMAX supplemented with 12% embryonic stem cell qualified fetal bovine serum (ATCC), 1% penicillin/streptomycin (HyClone); 10 mM HEPES (Invitrogen), 0.1 μg/ml hydrocortisone (Sigma-Aldrich), 10 μg/ml transferrin (Sigma-Aldrich), 0.4 ng/ml T3 (3,3,5-thio-iodo-thyronine) (Sigma-Aldrich), 10 pg/ml glucagon (Sigma-Aldrich), 1 ng/ml insulin (Sigma-Aldrich), 100 pg/ml epidermal growth factor (Sigma-Aldrich), and 200 pg/ml fibroblast growth factor 2 (Gibco)^48^.

Human Epidermal Melanocytes (HEMs) isolated from neonatal human skin were purchased from ScienCell Research Laboratories. HEMs were grown in poly-L-lysine-coated plates using MelM melanocyte medium (Cat. #2201, ScienCell Research Laboratories), as recommended by the supplier. Human cell lines were acquired as follows: 501mel from Yale University; 293T, A-375, 451Lu, MeWo, SK-MEL-2, and IGR-1 from American Type Culture Collection (ATCC); SK-MEL-85, SK-MEL-100, SK-MEL-103, SK-MEL-147, SK-MEL-197 were kindly provided by Alan Houghton (Memorial Sloan–Kettering Cancer Center, New York, NY, USA); WM278 from Meenhard Herlyn (Wistar Institute, Philadelphia, PA, USA) and WM239-derived 113/6-4L (designed as 4L) from Robert S Kerbel and William Cruz-Munoz^27^ (Sunnybrook Research Institute, Toronto, Canada); low passage melanoma short-term cultures (STCs), including 12-273BM, 10-230SC and 12-126BM were derived in Dr. Iman Osman laboratory as described^26^ and grown in DMEM with 10% fetal bovine serum (FBS), 1 mM sodium pyruvate, 4mM L-Glutamine, 25 mM d-Glucose, 1% non-essential amino acids (NEAA), 100 U/mL penicillin, and 100 mg/mL streptomycin. For epigenetic drug treatments, MeWo, 12-126BM, 10-230-SC, and SK-MEL-2 cells were treated with 5-aza-2′-deoxycytidine (Sigma) for 72 h, concentrations indicated in corresponding western blots (0, 1, 2.5, and 5 μM). Cell lines were validated using the STR method at ATCC. All STCs were matched to the respective donor. The identity of non-ATCC cells was validated using Promega’s Cell ID system (Cat.# G9500) by STR analysis^26^. All cell lines used in the study were tested negative for mycoplasma contamination prior to use in experiments. None were found contaminated. No commonly misidentified cell lines were used in the study.

### Melanoma DNA methylation profiles

DNA methylation from Illumina 450 K arrays and clinical data available for skin cutaneous melanoma patients were retrieved from The Cancer Genome Atlas-TCGA (https://portal.gdc.cancer.gov/, ref. ^50^).

### DNA methylation analyses

Genomic DNA was extracted from cell lines using QIAamp DNA Mini Kit (Qiagen) and then bisulfite treated using the EZ-DNA Methylation-Gold Kit (Zymo Research), according to manufacturers’ instructions. Methylation specific PCR (MSP) was performed using EpiTaq HS (Takara) and primers specific for methylated or unmethylated *NR2F2* Isoform 1 and Isoform 2 (Sequences available below, primers section). PCR amplified products were run on a 2% agarose gel for 20 min and visualized using the ImageQuant 300 Imager (Amersham Biosciences).

For DNA methylation profiling, genomic DNA was purified from samples using QIAamp DNA Mini Kit (Qiagen). DNA was quantified by fluorometric assay (Quant-iT Picogreen dsDNA, P7581, Life Technologies) by interpolating fluorescence signal with a standard curve of serialized dilutions (1000, 500, 250, 100, 50, 5, 2.5, 0 ng/μl). High molecular weight DNA was checked for all samples using a 1.3% agarose gel electrophoresis. Bisulfite conversion (EZ-DNA Methylation Kit ref. D5004, Zymo Research) was performed over 600 ng of high molecular weight genomic DNA for each sample. Bisulfite converted DNA was processed through the Infinium Methylation HD protocol in order to hybridize the samples on the Infinium HumanMethylation450 beadchip (Illumina), as described^51^. Fluorescent signal from the microarray was measured with a HiScan scanner (Illumina, Inc. San Diego) using iScan Control Software (V 3.3.29).

In addition to DNA methylation profiles generated for this study, raw data (IDATs) for human embryonic stem cell lines (GSE61461, ref. ^52^.), HEMs (GSE74877, ref. ^53^.) and nevi samples (GSE120878, ref. ^54^.) available at the Gene Expression Omnibus (GEO) public repository; primary and metastatic skin cutaneous melanoma samples retrieved from TCGA (https://portal.gdc.cancer.gov/, ref. ^50^.) and DNA methylation profiles of melanoma cell lines previously generated by Dr. Esteller’s group (GSE68379)^55^ were analyzed. All raw data were normalized using the minfi (v.1.19.10) package available for Bioconductor, under the R statistical environment (v.3.3.0), consisting background level adjustment and normalization among control probes included in the array (preprocessIllumina^56^). Methylation levels (*β*-value) were calculated as the ratio of methylated signal divided by the sum of methylated and unmethylated signals plus 100. All *β*-values with an associated *p* value greater or equal to 0.01 were removed from the analysis.

Using the manufacturer’s annotation for the Infinium HumanMethylation450, markers within the sexual chromosomes, as well as those for which a SNP is described within the last 10 bases of the oligonucleotide used to interrogate the CpG site, were removed from the analysis. Analysis of variance of markers was considered informative when the difference of methylation values among groups was |Δ*β*| ≥ 0.66 (|Δ*β*| ≥ 0.20 in primary vs. metastasis comparison) and the false discovery rate adjusted *p* value ≤ 0.01.

Validation of DNA methylation profiles was performed by bisulfite genomic sequencing (BSP), using EZ-DNA Methylation-Gold kit (Zymo Research, Orange, CA, USA; D5006) for DNA conversion and specific primers to amplify the regions of interest (Sequences available below, Primers section). Amplicons were cloned into the pGEM-T Easy Vector System I (Promega; A1360). Competent E. coli (DH5α strain) were transformed in LB-agar plates treated with ampicillin, X-Gal and IPTG. A minimum of eight clones were selected to calculate the methylation score. Plasmid purification for each clone was performed using the NucleoSpin 96 plasmid kit (Macherey-Nagel; 740625.24). Amplicon sequencing was performed using the 3730 DNA analyzer (Applied Biosystems; 3730S). Results were analyzed with BioEdit software and methylated cytosines were mapped using BSMap software.

### mRNA expression analysis

Total RNA was extracted and treated with DNAse using the RNAeasy Mini Kit (Qiagen) according to the manufacturer’s instructions. Reverse transcription was performed using the RETROscript (Applied Biosystems). Real-time quantitative PCR was carried out using Power Sybr Green PCR Master Mix (Applied Biosystems) and primers for *GAPDH*, *PPIA* and *HPRT1* (endogenous controls), *SNAIL* and *NR2F2* isoforms. Sequences for all primers are provided below. mRNA expression data from melanoma patients were retrieved from TCGA (https://portal.gdc.cancer.gov/, ref. ^50^.)

### RNA sequencing

Total RNA was extracted and treated with DNAse using the RNAeasy Mini Kit (Qiagen) according to the manufacturer’s instructions. Libraries were sequenced on an Illumina HiSeq 2500 sequencer. Sequencing results were demultiplexed and converted to FASTQ format using Illumina bcl2fastq software. The sequencing reads were aligned to the human genome (build hg19/GRCh37) using the splice-aware STAR aligner^57^. PCR duplicates were removed using the Picard toolkit [http://broadinstitute.github.io/picard/]. The HTSeq package^58^ was utilized to generate counts for each gene based on how many aligned reads overlap its exons. These counts were then normalized and used to test for differential expression using negative binomial generalized linear models implemented by the DESeq2 R package^59^.

### Protein expression analysis

Total protein was extracted using RIPA buffer (Pierce) with protease inhibitors (Roche) and phosphatase inhibitors (Roche). Cell lysates were resolved on NuPAGE 4–12% Bis-Tris Gels (Invitrogen) and transferred to PVDF membranes (Millipore). Membranes were blocked for 1 hour with 5% Blotting Grade Blocker (Bio-Rad) and probed with primary antibodies overnight at 4 °C. Membranes were then probed with peroxidase conjugated secondary antibodies. Peroxidase conjugated actin and tubulin (Sigma) were used as a loading control. List of antibodies is provided below.

### Subcellular fractionation

Whole cell pellets were fractionated using the NE-PER Nuclear and Cytoplasmic Extraction Reagents (ThermoFisher Scientific) according to manufacturer’s instructions. Nuclear and cytoplasmic protein fractions as well as whole cell lysates were subjected to immunoblotting. Purity of nuclear and cytoplasmic fractions was confirmed by probing for lamin-B (Santa Cruz) and tubulin (Sigma), respectively.

### Co-Immunoprecipitation

For native protein lysates, live cell cultures were washed with PBS and scraped. For cross-linked proteins, live cell cultures were fixed with 1% formaldehyde shaking for 10 min at room temperature. Fixation was stopped with 2.5 M Glycine by shaking for 5 min. Cells were washed with PBS and scraped. Pellets (native or cross-linked) were resuspended in lysis buffer (10 mM Tris/HCl pH 7.5, 150 mM NaCl, 0.5 mM EDTA, 0.5% NP-40, and protease/phosphatase inhibitors) and incubated on ice for 20 minutes, and then extracts were spun down. After spinning, supernatant was collected as whole cell extract and quantified by Lowry assay. One milligram of protein was combined with beads and incubated rotating overnight at 4 °C. On the following day, protein extract was removed and beads were washed three times with wash buffer (10 mM Tris/HCl pH 7.5, 150 mM NaCl, 0.075% NP-40, and protease/phosphatase inhibitors). Beads were boiled in 2X loading buffer with DTT for 10 minutes at 95 °C. For pull down of GFP, GFP Trap (ChromoTek) beads were washed with PBS and lysis buffer before incubation with whole cell extracts. For pull down of NR2F2 Isoform 1, Dynabeads Protein A (Invitrogen) were cross-linked to NR2F2-Isoform 1 antibody (cat# 41859 Abcam, or Active Motif catalog # 61214). Beads were blocked overnight in 5% BSA at 4 °C, then washed and incubated with 5 μg antibody or AffinPure Goat Anti-Mouse IgG (Jackson) control for 2 h at 4 °C. Antibody was fixed to beads during a 30 min incubation at room temperature with 5 mM BS3 (bis(sulfosuccinimidyl)suberate) (ThermoFisher Scientific). Fixation was stopped by addition of 1 M Tris/HCl pH 7.5. Antibody-fixed beads were washed with lysis buffer before overnight incubation with whole cell extracts.

### Short hairpin interference and ectopic expression assays

Lentiviral vectors were used for knockdown or ectopic expression of NR2F2. A list of plasmids is provided in Supplementary Information. 293T cells were used to generate the lentiviral particles. 293T cells were transfected with 12 μg of plasmid of interest, 8 μg viral packaging plasmid (psPAX2), and 4 μg viral envelope plasmid (pMD2.G) using Lipofectamine 2000 (Invitrogen) in OPTI-MEM (Gibco). Lentiviral particle-containing supernatants were collected 48 h after transfection and filtered using 0.45 μm filters. Melanoma cell lines in medium supplemented with 10% heat inactivated FBS were then infected with lentiviral supernatants for 6 h in the presence of 8 μg/ml polybrene (InvivoGen). Medium containing virus was replaced with medium supplemented with 5% heat inactivated FBS. Positive cells were selected and maintained in 2.5 μg/ml puromycin-contained medium or sorted for mCherry/GFP positive cells at the NYU Langone Flow Cytometry Core, depending on the vector.

### Chromatin Immunoprecipitation (ChIP) sequencing

ChIP-IT High Sensitivity kit (Active Motif) was used according to the manufacturer recommendations, using a ChIP grade NR2F2 antibody specific for isoform 1 (catalog # 61214, Active Motif). ChIP-Seq libraries were generated using standard Illumina kit. Libraries were sequenced on an Illumina HiSeq 2500 sequencer. Sequencing results were demultiplexed and converted to FASTQ format using Illumina bcl2fastq software. Reads were aligned to the human genome (build hg19/GRCh37) with Bowtie 2^60^ using local alignment. Only confidently mapped reads (mapping quality > 20) were retained. Duplicate reads were discarded using Picard [http://broadinstitute.github.io/picard/]. MACS^61^ was utilized to perform broad peak calling for each replicate with a *q*-value cutoff of 0.01. Bedtools^62^ was used to identify peaks that were called in either SCR, shA, or both conditions and their binding profiles were visualized as histograms using Deeptools^63^. Regions of maximum central enrichment in ChIP-seq peaks were identified by CentriMo^64^ (MEME-suite^65,66^). Genes associated with NR2F2-ChIP-seq peaks were identified with GREAT—Genomic Regions Enrichment of Annotations Tool^35^—using standard parameters. Differentially enriched transcription factor motifs on NR2F2-bound sites that are up- or down-regulated dependent on NR2F2-iso2 expression were discovered with HOMER^38^ motif analyses tools. Venn diagrams were generated with Biovenn.nl^67^.

### Proliferation assay

Cells were seeded at low density in 24-well (short-term cultures) and 96-well plates. At time points of 1–7 days post seeding, cells were fixed with 15% glutaraldehyde and stained with 0.5% crystal violet. Crystal violet was eluted with 15% acetic acid and measured at 590 nm using a FlexStation 5 Plate Reader.

### Soft agar assay

In 12-well plates, 1 ml base layer of 0.5% Agar (BD Difco Agar Noble) in 2× complete medium was plated and allowed to gel. A layer of single cell suspension in 0.35% agar in 2× complete medium was then plated on top of the base layer and allowed to gel. Five hundred microliters of complete medium was added and replenished to prevent desiccation. Colony formation was monitored and imaged with the EVOS FL Cell Imaging System, and was imaged using the ArrayScan VTI (Cellomics) and analyzed using the Morphology Explorer Bioapplication V4 Thermo Cellomics HCS Studio at the High Throughput Biology Core at NYU Langone Medical Center. Only objects captured with an area greater than, or equal to, 5000 pixels were used for analysis in each experiment. As indicated in the respective figure legends, all colony formation assays were analyzed at least 21 days post seeding.

### Melanosphere formation assay

Cells were plated in hESCM4 media [70% human embryonic stem cell hES media (80% DMEM/F12 (Invitrogen), 20% Knockout Serum replacement (Invitrogen), 0.1 mM beta-mercaptoethanol, 1mM L-glutamine (Fisher), 1X MEM amino acids (Corning), 1× penicillin–streptomycin (HyClone)); 30% conditioned media (from mouse embryonic fibroblasts cultured in hES media for 24 h) and 4 ng/ml basic fibroblast growth factor (R&D Systems)] in 96-Well Ultra Low Attachment Spheroid Microplates (Corning). Spheres were imaged using the ArrayScan VTI (Cellomics)and analyzed using the Morphology Explorer Bioapplication V4ThermoCellomicsHCS Studio at the High Throughput Biology Core at NYU Langone Medical Center.

### Mouse xenograft models

To evaluate tumor growth, 1 × 10^6^ cells stably transduced with luciferase/mCherry and control, shNR2F2-Iso2 or NR2F2 ectopic expression were subcutaneously injected in the flank of NOD.Cg-Prkdc^scid^ Il2rg^tm1Wjl^/SzJ (NSG) mice (The Jackson Laboratory). Twice a week, mice were weighed and tumors were measured using a two-dimensional caliper to calculate tumor volume (volume = (*π*/6) × length × wide^2^). To follow metastasis development, primary tumors were surgically removed when they reached 500–600mm^3^. Metastatic ability was also monitored upon ultrasound imaging-guided intracardiac injection of 250,000 (4L cells) or 100,000 (12–273BM, WM278, and MeWo cells) shNR2F2-Iso2 or NR2F2 overexpressing cells, or controls in athymic nude (nu/nu) or NSG mice (The Jackson Laboratory). In all experiments, metastatic burden was followed measuring radiance once a week using an In Vivo Imaging System (IVIS) at the NYU Langone’s Experimental Animal and Exposure Core. After euthanasia, organs were harvested and imaged in a M165 Fluorescent Stereo Microscope (Leica) and fixed in 10% formalin for 48 h. Metastatic lesions on samples were examined macroscopically and counted microscopically by a pathologist following H&E staining. Animal experiments were conducted in accordance with guidelines set forth by the Institutional Animal Care and Use Committee (IACUC) of NYU (protocol # 120405-02). The maximal tumor size/burden permitted by our institutional review board is 1500 mm^3^, and we confirm that the maximal tumor size/burden was not exceeded.

### Mouse housing conditions

Animals are housed in an AAALAC-accredited research facility. Rodent housing rooms are maintained at a temperature range of 21–23 °C with a humidity range of 30–70% and a 12:12-h light:dark cycle. Housing room air exchange rates are set at 10–15 air changes per hour. Mice are provided ad libitum autoclaved water and irradiated feed (5058 irradiated rodent chow, LabDiet, St. Louis, MO). Water is filtered prior to autoclaving. All mice are housed in autoclaved individually ventilated caging (Tecniplast, West Chester, PA) at 50–70 cage-volume air changes hourly. Cages are filled with 1/8 –in. corncob bedding (Bed-o-Cobs, Anderson, Maumee, OH) and each rodent cage receives nesting material (Nestlet, Ancare Corp., Bellmore, NY). Colony health surveillance is performed quarterly using a combination of dirty bedding sentinels and exhaust air dust testing. The following viral, bacterial, and parasitic pathogens are excluded from the rodent colony including mouse parvovirus (1–5), minute virus of mice, mouse hepatitis virus, mouse norovirus, Theiler’s murine encephalomyelitis virus, epizootic diarrhea of mice, Sendai virus, pneumonia virus of mice, reovirus, *Mycoplasma pulmonis*, lymphocytic choriomeningitis virus, mouse adenovirus, ectromelia, K virus, polyomavirus, mouse cytomegalovirus, hantavirus, *Encephalitozoon cuniculi*, CAR Bacillus, mouse thymic virus, lactate dehydrogenase elevating virus, *Clostridium piliforme*, Helicobacter, fur mites and pinworms.

### Statistical analysis

Details are provided for each experiment. In brief, at least three independent experiments were performed to confirm colony and sphere formation results. At least 9 mice per group were used for mouse model assays, as detailed in each experiment. Statistical significance between groups were analyzed using Student’s t test, ANOVA, Fisher’s exact test or Mann-Whitney test, as denoted in each experiment based on number of groups or parameters being tested. Correlations were evaluated using Spearman correlation test. *p* values < 0.05 were considered significant. Analyses were performed with Prism 9 software.

### Primers

qPCR-GAPDH (CAAGATCATCAGCAATGCCT, AGGGATGATGTTCTGGAGAG). qPCR-PPIA (ATGGTCAACCCCACCGTGT, TCTGCTGTCTTTGGGACCTTG). qPCR-HPRT1 (TGACACTGGCAAAACAATGCA, GGTCCTTTTCACCAGCAAGCT). qPCR-NR2F2-Iso1 (GGAGGAACCTGAGCTACAC, TATCCGGACAGGTACGAGT). qPCR-NR2F2-Iso2 (CCAAACTAAAGGAGAGTTATTCCA, GTACGAGTGGCAGTTGAGG). qPCR-SNAIL (CACTATGCCGCGCTCTTTC, GGTCGTAGGGCTGCTGGAA). MSP-NR2F2-Iso2-Unmethylated (TGAGGGAAGTTTGTTTGTTAGTTTGT, CCACCAACAACTATAAACAATATTAC). MSP-NR2F2-Iso2-Methylated (CGAGGGAAGTTTGTTTGTTAGTTTGC, CCGCCAACAACTATAAACGATATTAC). NR2F2-Iso2-Bseq (ATTATTTGGGGAGATTTGAGT, CCATATATTAAACTCTCTCAACCTT).

### Plasmids

Melanoma cells were transduced with a lentiviral vector containing both mCherry and firefly luciferase (luc)^68^ to track cells in vitro and in vivo, respectively. Cells stably expressing mCherry and luciferase were used for knockdown and ectopic expression experiments. NR2F2-isoform 2 knockdown was performed by short hairpin interference using two shRNAs: shNR2F2-Iso2-A (HT132379B, Origene) and shNR2F2-Iso2-B (HC133711B, Origene). NR2F2-isoform 1 knockdown was performed by short hairpin interference using two shRNAs: shNR2F2-Iso1-X and shNR2F2-Iso1-Y (HSH018031, GeneCopeia). Non-targeting 29-mer Scrambled shRNA Cassette in pGFP-C-shLenti Vector (TR30021, Origene) was used as control. Lentiviral vectors were also used for NR2F2 ectopic expression: NR2F2 Isoform 1 (EX-C0221-Lv205, GeneCopoeia) and NR2F2 Isoform 2 (RC226609L2, Origene). pEZ-Lv205 (EX-NEG-Lv205, GeneCopoeia) or pLenti-C-mGFP (PS100071, Origene) were used as control vectors.

### Antibodies

NR2F2 Isoform 1 (41859, Abcam for western blot (1:1000)); 61214, Active Motif for ChIP (5 μL per sample), NR2F2 Isoform 2 (Millipore custom antibody (1:1000)), SNAIL (3879, Cell Signaling Technology (1:1000)), Actin (A3854, Sigma (1:50,000)), Lamin-B (sc6217, Santa Cruz (1:50,000)), Alpha-tubulin (T9026, Sigma (1:50,000)). Rabbit secondary (Sigma, A0545 (1:20,000)), mouse secondary (Sigma, A9044 (1:20,000)), rat secondary (Millipore, AP136P (1:20,000)), and goat secondary (Sigma, A5420 (1:20,000)).

### Reporting summary

Further information on research design is available in the Nature Portfolio Reporting Summary linked to this article.

## Supplementary information

Supplementary Information

Description of Additional Supplementary Files

Supplementary Data 1

Supplementary Data 2

Reporting Summary

## Data Availability

The methylation array data generated in this study have been deposited in GSE102542 and GSE213392 (methylation arrays) with unrestricted access. The sequencing data have been deposited in the GSE102554 superseries, which includes subseries GSE102552 (ChiP-seq data) and GSE102553 (RNA-seq data) with unrestricted access. All data are available in the article, supplementary information and source data. Source data are provided with this paper.

## Source data

Source Data
