# Supplementary material for: An epigenetic switch controls an alternative NR2F2 isoform that unleashes a metastatic program in melanoma
Source: Nat Commun. 2023 Apr 4;14:1867. doi: 10.1038/s41467-023-36967-2 (PMC10073109; doi:10.1038/s41467-023-36967-2)
Supplement: Supplementary file 1 — Supplementary Information [file 41467_2023_36967_MOESM1_ESM.pdf]

a

NCCs vs. melanocytes	Metastasis vs. Primary melanoma	Shared
Hypermethylated (n=1188)	Hypermethylated (n=784)	41
Hypomethylated (n=1373)	Hypomethylated (n=445)	14
Hypermethylated (n=1188)	Hypomethylated (n=445)	0
Hypomethylated (n=1373)	Hypermethylated (n=784)	1

β value

0 1

b

41 hypermethylated CpGs in NCCs and melanoma metastasis

CpG ID	Chr	Coordinate	Genes	NCCs	Melanocytes	diff	p value	Primary	Metastasis	diff	p value
cg02025435	16	89986317	*MC1R*	1.0	0.2	-0.8	4.90416E-06	0.5	1.0	0.4	9.04653E-13
cg01511465	16	89986185	*MC1R*	1.0	0.1	-0.9	3.31277E-09	0.6	1.0	0.4	2.77595E-13
cg06475006	16	89985975	*MC1R*	1.0	0.2	-0.7	1.21266E-06	0.5	0.9	0.3	6.50386E-12
cg06297571	12	132270829	*SFRS8*	0.9	0.0	-0.9	9.89803E-11	0.4	0.7	0.3	8.3561E-11
cg07009334	6	1410489		0.9	0.2	-0.7	3.49868E-06	0.5	0.8	0.3	2.04064E-05
cg17044311	10	101542983	*ABCC2*	0.9	0.1	-0.7	1.70746E-07	0.5	0.8	0.3	4.45334E-12
cg01768446	16	89982419		1.0	0.1	-0.8	6.4127E-09	0.5	0.7	0.3	7.90363E-09
cg19458233	3	100148679	*LNP1*	0.9	0.0	-0.9	5.83777E-12	0.5	0.8	0.3	0.000116675
cg16613990	16	89982618		0.9	0.1	-0.8	2.16979E-09	0.6	0.8	0.2	2.09077E-09
cg02922150	16	1514587	*CLCN7;CLCN7*	0.9	0.2	-0.7	9.70742E-07	0.5	0.8	0.2	1.21004E-11
cg12198090	12	132270765	*SFRS8*	0.9	0.2	-0.7	1.81025E-06	0.6	0.8	0.2	1.29253E-08
cg15770585	7	4762742	*FOXK1*	1.0	0.0	-0.9	6.85955E-13	0.6	0.8	0.2	4.72437E-05
cg09867143	16	1514413	*CLCN7;CLCN7*	0.8	0.2	-0.7	2.15705E-07	0.6	0.8	0.2	5.45373E-12
cg14583973	4	3374767	*RGS12;RGS12;RGS12*	0.9	0.1	-0.8	2.18574E-05	0.5	0.8	0.2	7.1024E-05
cg22094306	2	131834952	*FAM168B*	0.8	0.1	-0.7	6.57535E-09	0.6	0.8	0.2	3.93509E-08
cg03699749	20	60869595	*OSBPL2;OSBPL2*	0.9	0.0	-0.9	9.40279E-12	0.4	0.6	0.2	8.64547E-07
cg20957542	22	43529321	*MCAT;MCAT*	0.8	0.1	-0.7	2.73133E-11	0.4	0.6	0.2	3.49224E-12
cg14414203	14	23315249	*MMP14*	0.9	0.2	-0.7	6.64945E-05	0.6	0.8	0.2	0.000183675
cg13496793	9	129645969	*ZBTB34*	0.9	0.1	-0.8	1.07848E-08	0.6	0.8	0.2	4.47707E-06
cg01198184	14	103433908	*CDC42BPB*	0.9	0.1	-0.8	3.50269E-11	0.4	0.6	0.2	1.54669E-07
cg09951047	9	97848306	*C9orf3;MIR24-1*	0.9	0.1	-0.7	1.17201E-07	0.5	0.7	0.2	3.65993E-08
cg03647436	10	134477441	*INPP5A*	1.0	0.1	-0.9	1.31383E-06	0.5	0.7	0.2	5.6155E-07
cg10914815	13	99148667	*STK24;STK24*	0.8	0.1	-0.7	4.59109E-08	0.3	0.5	0.2	2.18322E-06
cg10397930	11	109959298		0.9	0.1	-0.8	3.6357E-07	0.5	0.7	0.2	1.24517E-06
cg18773129	22	19938916	*COMT;COMT;COMT*	0.9	0.1	-0.9	1.82073E-10	0.4	0.6	0.2	0.002023505
cg11435167	19	1588449	*MBD3*	0.9	0.1	-0.9	6.77279E-13	0.5	0.6	0.2	1.18356E-09
cg07155331	12	125500511	*BRI3BP*	0.9	0.1	-0.8	1.34536E-09	0.5	0.7	0.2	0.000293968
cg09121485	7	6218888	*CYTH3*	0.9	0.2	-0.7	4.19519E-08	0.7	0.8	0.2	1.48573E-10
cg03562952	5	1499204	*LPCAT1*	0.9	0.2	-0.8	1.29539E-07	0.5	0.7	0.2	3.52919E-07
cg16871520	3	52442033	*BAP1*	0.9	0.2	-0.7	4.47327E-10	0.5	0.7	0.2	4.65478E-07
cg16283158	14	103433189	*CDC42BPB*	0.9	0.1	-0.8	3.08864E-07	0.7	0.8	0.2	8.59925E-13
cg05904317	4	120518737	*PDE5A;PDE5A;PDE5A*	0.9	0.1	-0.8	1.65271E-10	0.4	0.6	0.2	3.63482E-07
cg25030694	2	134329911		0.9	0.2	-0.7	9.16219E-08	0.6	0.7	0.2	4.35E-08
cg14873022	4	146841701	*ZNF827*	0.8	0.0	-0.8	4.91835E-08	0.3	0.5	0.2	1.96966E-07
cg24211657	17	76886742	*TIMP2*	0.9	0.1	-0.8	3.96996E-09	0.3	0.5	0.2	2.23063E-07
cg08240529	2	37571401	*QPCT*	0.9	0.2	-0.7	3.47555E-07	0.6	0.8	0.2	4.52068E-07
cg19612114	14	103433138	*CDC42BPB*	0.9	0.2	-0.7	3.12931E-07	0.7	0.8	0.2	1.40451E-11
cg25989745	9	12693326	*TYRP1*	0.8	0.1	-0.7	8.88522E-09	0.6	0.8	0.2	6.56294E-05
cg00163372	8	128752988	*MYC*	0.8	0.0	-0.8	1.13757E-08	0.3	0.5	0.2	0.016939887
cg24721056	18	11818701	*GNAL;GNAL;GNAL*	0.8	0.2	-0.7	6.59852E-06	0.4	0.5	0.2	1.33326E-06
cg01916088	6	41528531	*FOXP4;FOXP4;FOXP4*	0.8	0.0	-0.8	2.5837E-06	0.1	0.3	0.2	0.026830917

c

14 hypomethylated CpGs in NCCs and melanoma metastasis

CpG ID	Chr	Coordinate	Genes	NCCs	Melanocytes	diff	p value	Primary	Metastasis	diff	p value
cg05108467	15	96869132	*NR2F2*	0.1	0.8	0.7	1.45523E-06	0.6	0.2	-0.3	7.33737E-08
cg03018496	15	96868989	*NR2F2*	0.1	0.9	0.7	1.07305E-05	0.7	0.4	-0.3	1.8228E-07
cg26593946	15	96869043	*NR2F2*	0.1	0.9	0.8	3.7246E-07	0.6	0.3	-0.3	7.30956E-07
cg09969043	15	96868971	*NR2F2*	0.1	0.8	0.7	1.02828E-05	0.5	0.2	-0.3	5.76819E-08
cg17826834	15	96868857	*NR2F2*	0.2	0.9	0.7	5.98012E-06	0.6	0.3	-0.3	3.61954E-07
cg20245757	17	46663759		0.0	0.9	0.8	1.65151E-07	0.6	0.4	-0.2	0.000182355
cg17719317	4	6909606		0.1	0.9	0.8	9.89475E-09	0.5	0.3	-0.2	0.003165727
cg24811290	4	159092553	*FAM198B*	0.2	0.9	0.7	7.69637E-07	0.5	0.3	-0.2	1.30675E-06
cg04883026	11	66487938	*SPTBN2*	0.2	0.9	0.7	1.03279E-06	0.5	0.4	-0.2	0.008358493
cg22957135	14	33409651	*NPAS3*	0.1	0.8	0.7	2.46678E-08	0.3	0.1	-0.2	2.04732E-07
cg08325519	12	130571246		0.2	0.9	0.7	6.5131E-10	0.4	0.2	-0.2	0.000171311
cg27164797	6	57038231	*BAG2*	0.1	0.9	0.8	5.67013E-08	0.7	0.6	-0.2	9.75422E-06
cg00436496	15	96885119		0.2	0.9	0.7	1.51868E-05	0.6	0.5	-0.2	0.000105765
cg03159396	6	50808813	*TFAP2B*	0.1	0.8	0.7	3.90715E-09	0.7	0.5	-0.2	0.008125247

Suppl. Fig. 1. Differentially methylated CpGs between Neural crest cells (NCCs) vs. melanocytes, or in TCGA metastatic vs. primary melanoma samples. a. Integration analysis was performed to identify hypermethylated or hypomethylated CpGs between NCCs vs. melanocytes and metastasis vs. primary melanoma samples. Forty-one hypermethylated **(b**) and fourteen hypomethylated (**c**) CpGs shared by NCCs and metastasis were identified. Top 5 hypomethylated CpGs were located in the Nuclear Receptor Subfamily 2 Group F Member 2 (*NR2F2*) promoter region. Methylation scores (Beta values) ranging from 0 (unmethylated, green) to 1 (complete methylated, red) were obtained from HumanMethylation450K arrays. Differential methylation between groups and p values (unpaired t-test) are depicted. Of note, only one hypomethylated CpG (cg08426157, *HDAC9* gene body) and no hypermethylated CpGs were shared between NCCs and primary melanoma samples (**a**).

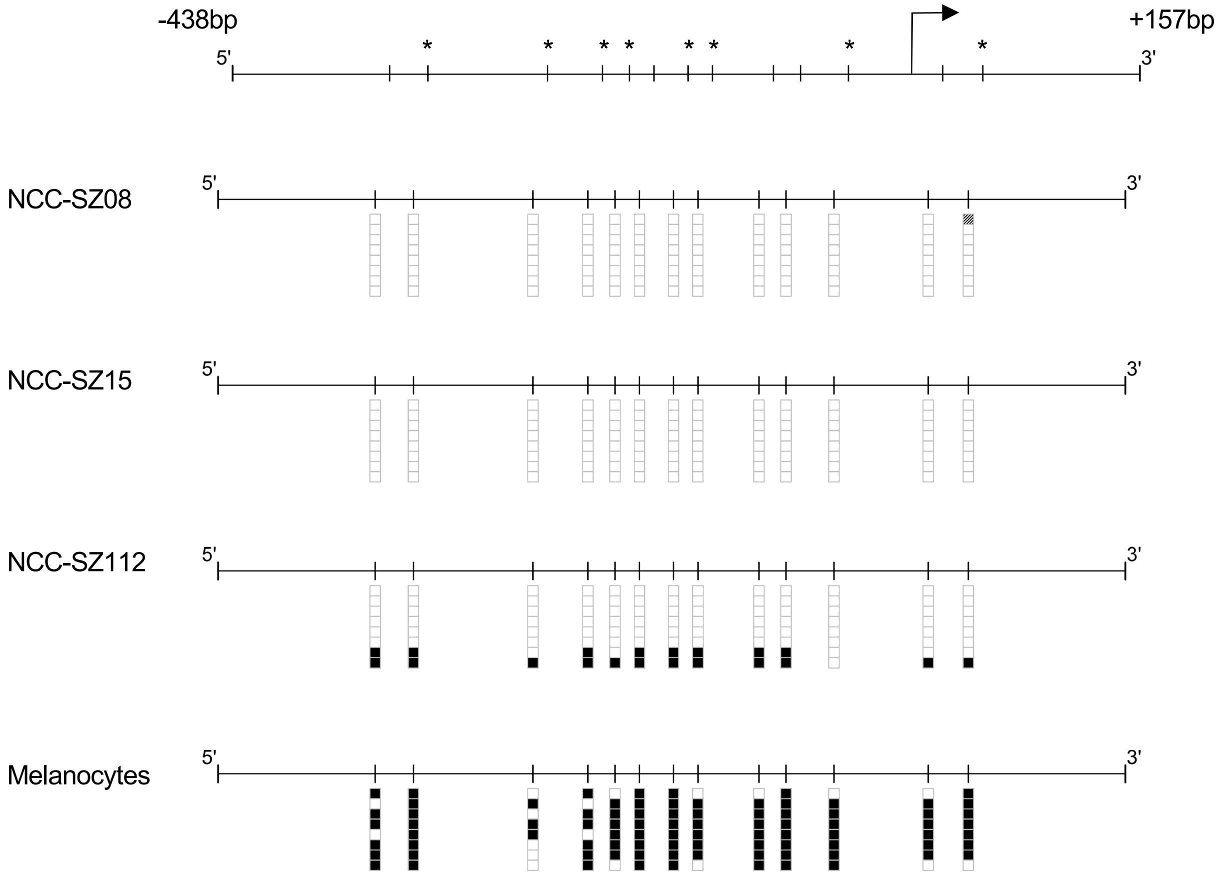

Suppl. Fig. 2. *NR2F2*-Isoform 2 methylation profiles in neural crest cells and melanocytes. Bisulfite sequencing of the region around the *NR2F2*-Iso2 transcription start site (-438bp to +157bp) in neural crest cells and melanocytes, validating the data generated by DNA methylation arrays. CpGs are represented as white (unmethylated) or black (methylated) squares. *: CpGs interrogated in the 450K methylation array; arrow: Transcription start site (TSS).

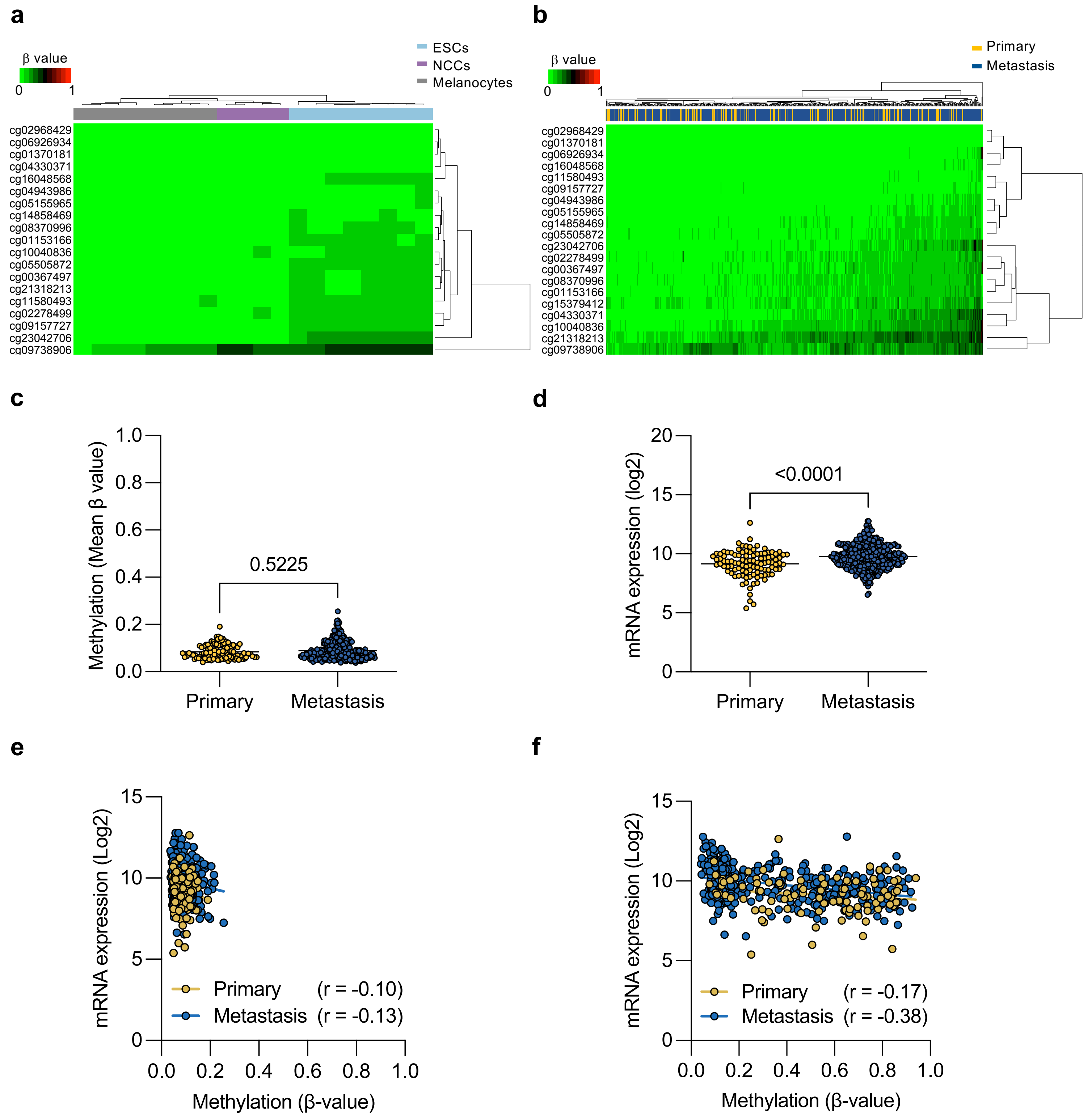

Suppl. Fig. 3. *NR2F2*-Isoform 1 methylation and expression in ESCs, NCC, melanocytes and during melanoma progression. Absence of methylation in the CpG island around the transcription start site (TSS) of isoform 1 in embryonic stem cell lines (ESCs), neural crest cells (NCCs) and melanocytes (**a**); and in primary and metastatic melanoma (**b, c**). Methylation scores (Beta values) ranging from 0 (unmethylated, green) to 1 (completely methylated, red) were obtained from HumanMethylation450K arrays. The depicted CpGs are located from -261 to 1719pb to the *NR2F2*-Iso1 TSS. *NR2F2*-Iso1 mRNA expression of primary and metastatic TCGA melanoma samples (**d,** two tailed Mann-Whitney test, bars represent min. and max.). Correlation between *NR2F2*-Iso1 expression and methylation around the TSS of isoform 1 (**e,** Spearman's rank correlation) and of isoform 2 (**f,** Spearman's rank correlation) in TCGA melanoma samples. Source data are provided as a Source Data file.

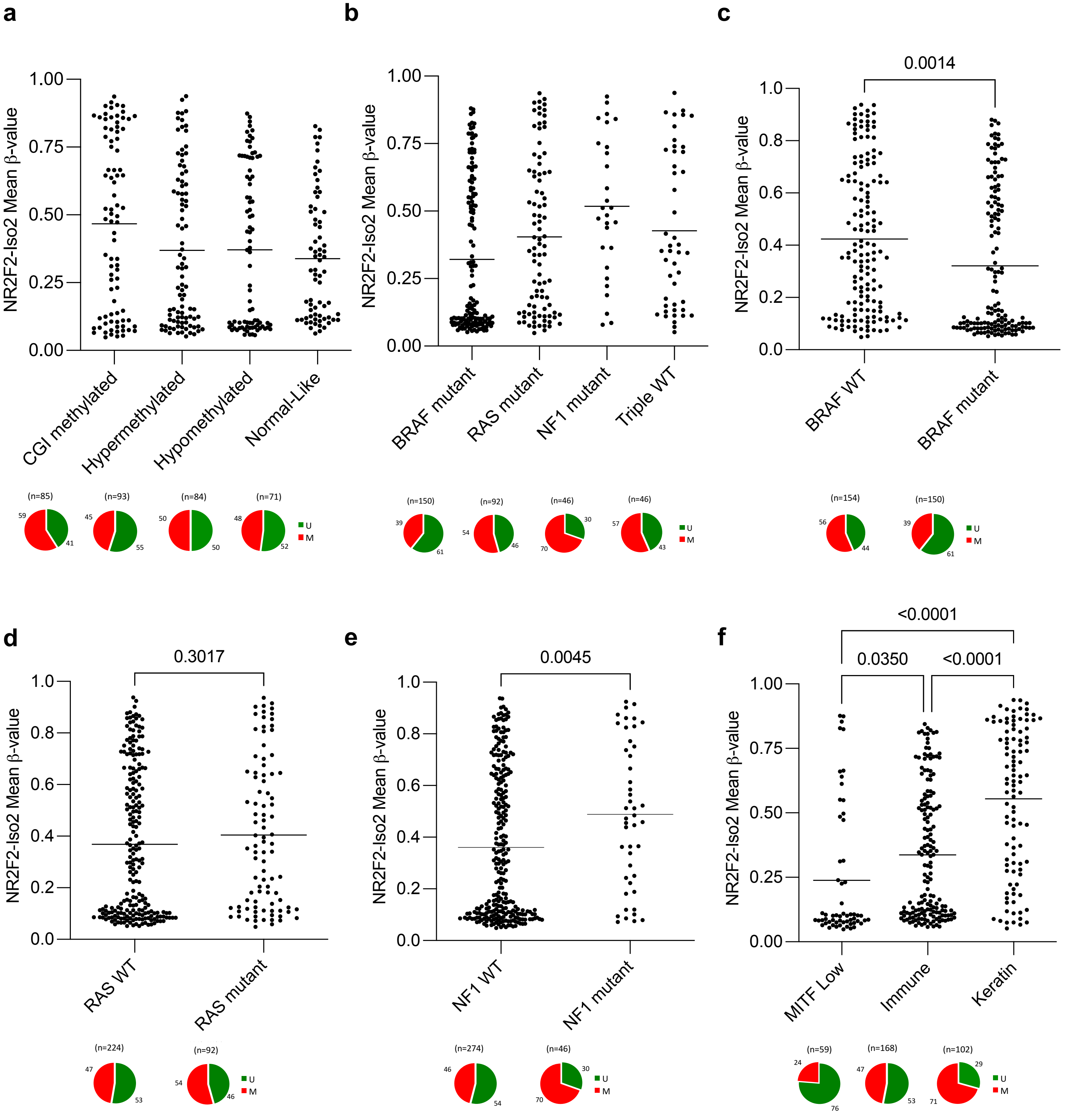

Suppl. Fig. 4. *NR2F2*-Iso2 methylation in different melanoma subtypes. (a-f). *NR2F2* isoform 2 methylation status was evaluated across the distinct subtypes defined by The Cancer Genome Atlas Network (Cell, 2015). *NR2F2*-Iso2 methylation (mean Beta-values) in patients classified by DNA methylation clusters (**a**); patients harboring *BRAF, RAS (NRAS, KRAS or HRAS)* or *NF1* hot-spot mutations, and triple wild-type (**b**); additional comparisons of NR2F2-Iso2 methylation levels between *BRAF* mutant and wt (**c,** unpaired two-tailed T-test), *RAS (NRAS, KRAS or HRAS)* mutant and wild-type (**d,** unpaired two-tailed T-test) and *NF1* mutant and wt (**e,** unpaired two-tailed T-test)**;** Comparison of *NR2F2*-Iso2 methylation levels among TCGA-defined transcriptomic subclasses named “MITF low”, “immune” and “keratin” (**f,** Two-way ANOVA). U: unmethylated, green; M: methylated, red. Bars represent the mean. Source data are provided as a Source Data file.

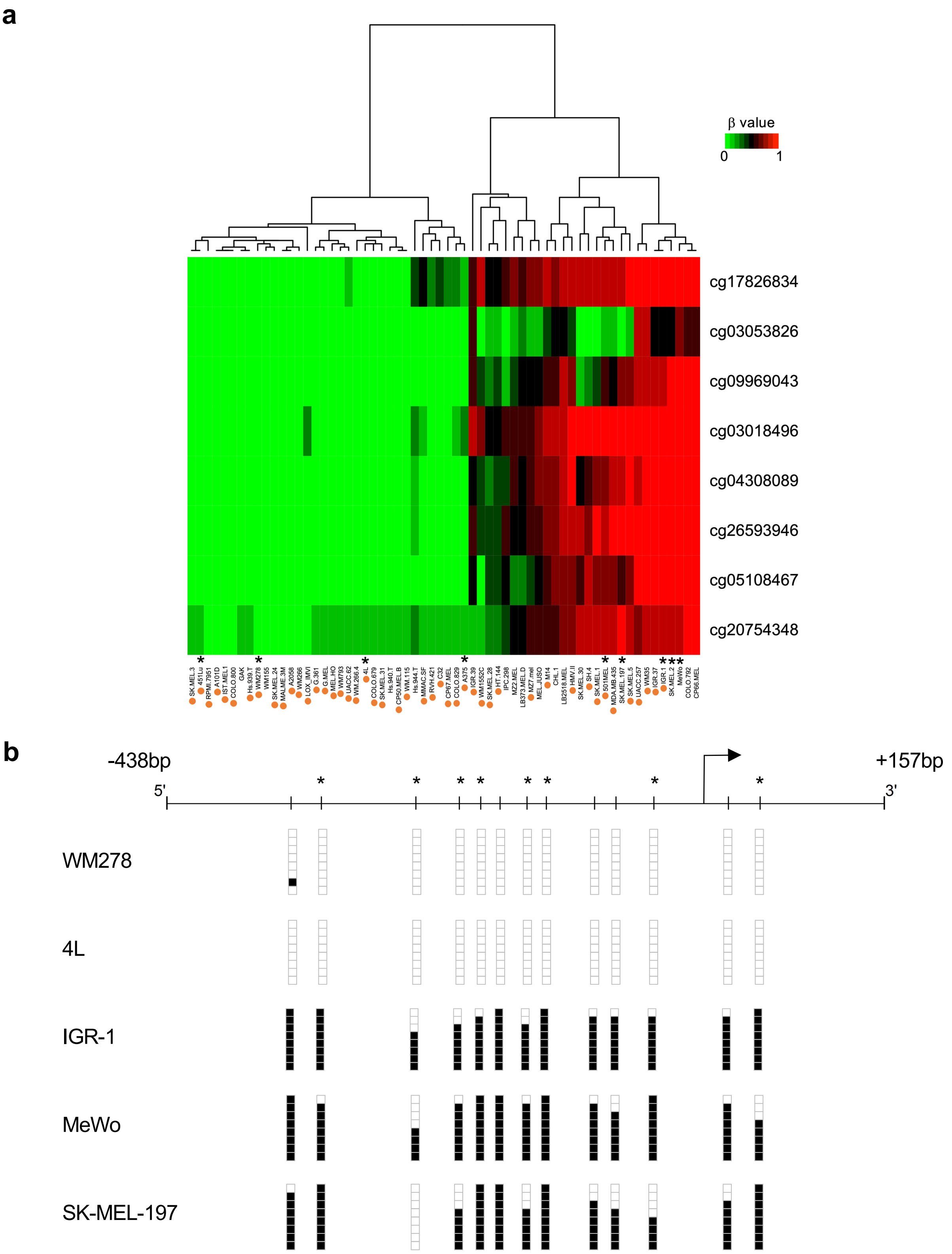

Suppl. Fig 5. *NR2F2*-Iso2 methylation profile in a set of melanoma cell lines. a. Unsupervised clustering analysis of the differentially methylated CpGs located in the *NR2F2-iso2* promoter region detected clusters of hypomethylated and hypermethylated cell lines. Methylation scores (Beta values) ranging from 0 (unmethylated, green) to 1 (complete methylated, red) were obtained from HumanMethylation450K arrays. (*): cell lines used for RT-PCR and WB analyses (Fig. 2A, B). Orange circle: BRAF mutant cell lines. **b.** Bisulfite sequencing of the region around the *NR2F2*-Iso2 TSS (-438bp to +157bp) in WM278, 4L, IGR-1, MeWo and SK-MEL-197 melanoma cell lines, validating the data generated by DNA methylation arrays. CpGs are represented as white (unmethylated) or black (methylated) squares. *: CpGs interrogated in the 450K methylation array; arrow: Transcription start site (TSS).

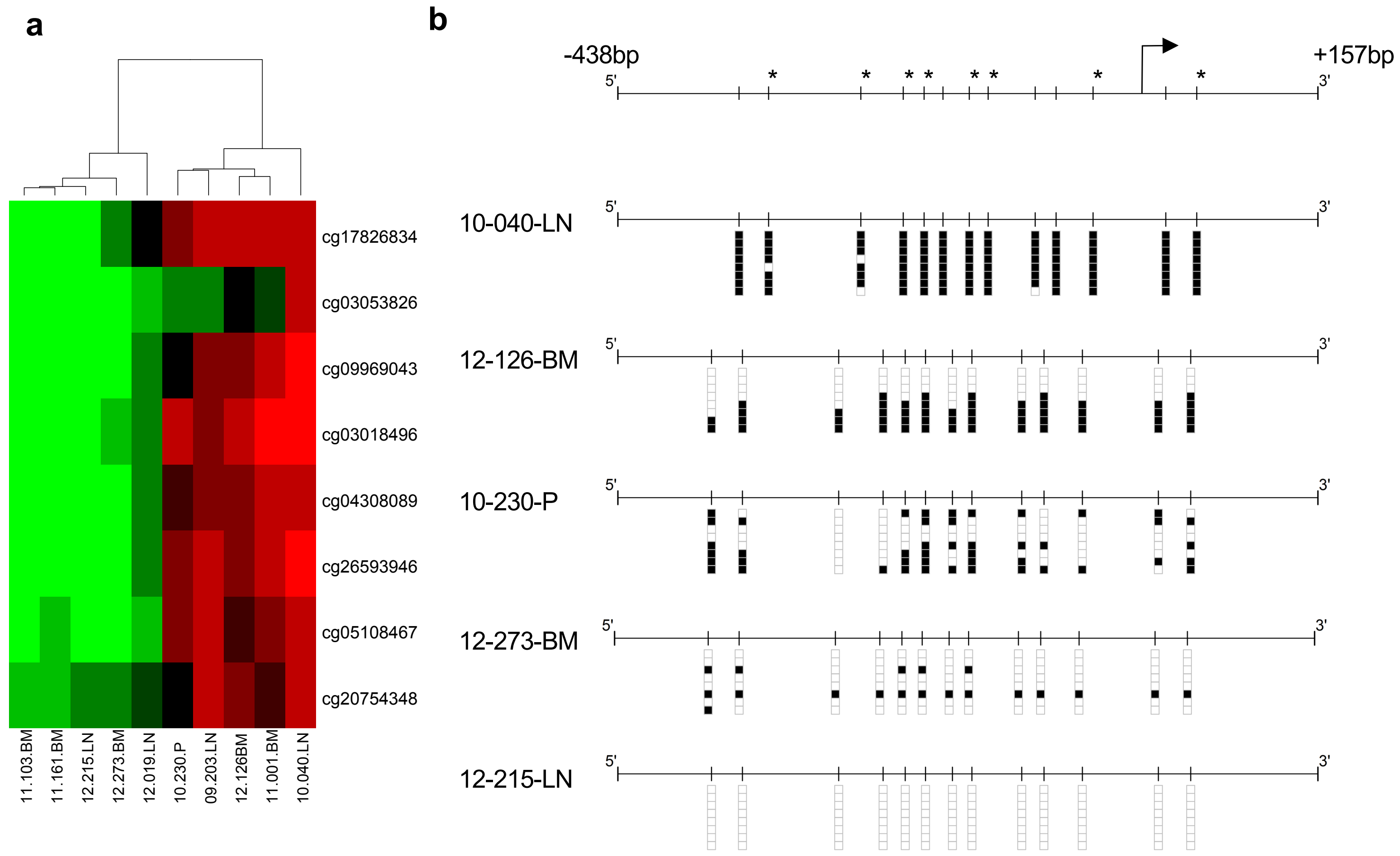

Suppl. Fig 6. *NR2F2*-Isoform 2 methylation profiles in melanoma patient-derived short term cultures (STC). a. Methylation scores (Beta values) ranging from 0 (unmethylated, green) to 1 (completely methylated, red) were obtained from HumanMethylation450K arrays. The depicted CpGs are located from -300 to +63pb to the *NR2F2*-Iso2 TSS. **b**. Bisulfite sequencing of the region around the *NR2F2*-Iso2 TSS (-438bp to +157bp) in STCs, validating the data generated by HumanMethylation450K arrays. CpGs are represented as white (unmethylated) or black (methylated) squares. *: CpGs interrogated in the HumanMethylation450K arrays; arrow: Transcription start site (TSS).

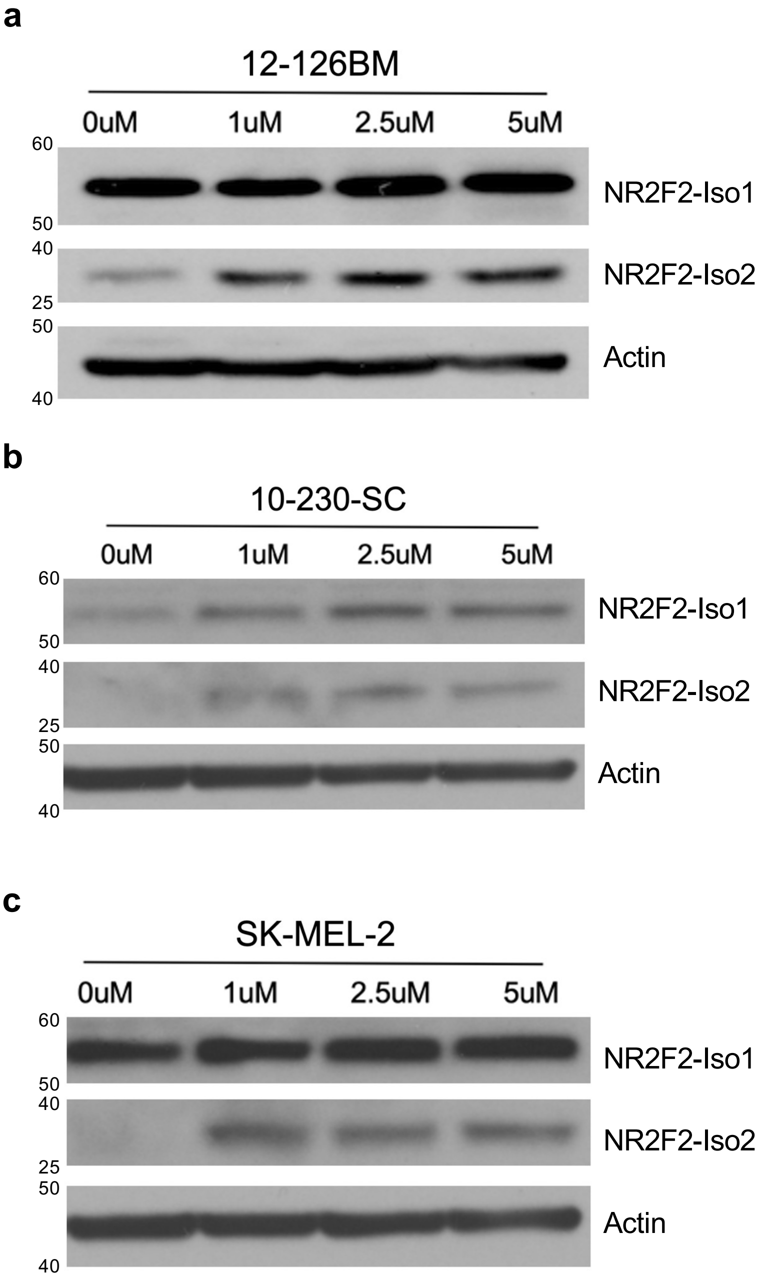

Suppl. Fig 7. Global demethylation of hypermethylated *NR2F2*-Iso2 melanoma cells restores NR2F2-Iso2 expression. Treatment of 12-126BM (**a**), 10-230SC (**b**), and SK-MEL-2 (**c**) hypermethylated cells with DNA demethylating agent 5'-aza-2'-deoxycytidine (1, 2.5, and 5uM aza, 72h) restores NR2F2-Iso2 protein expression evaluated by immunoblot. Experiments have been conducted twice. Source data are provided as a Source Data file.

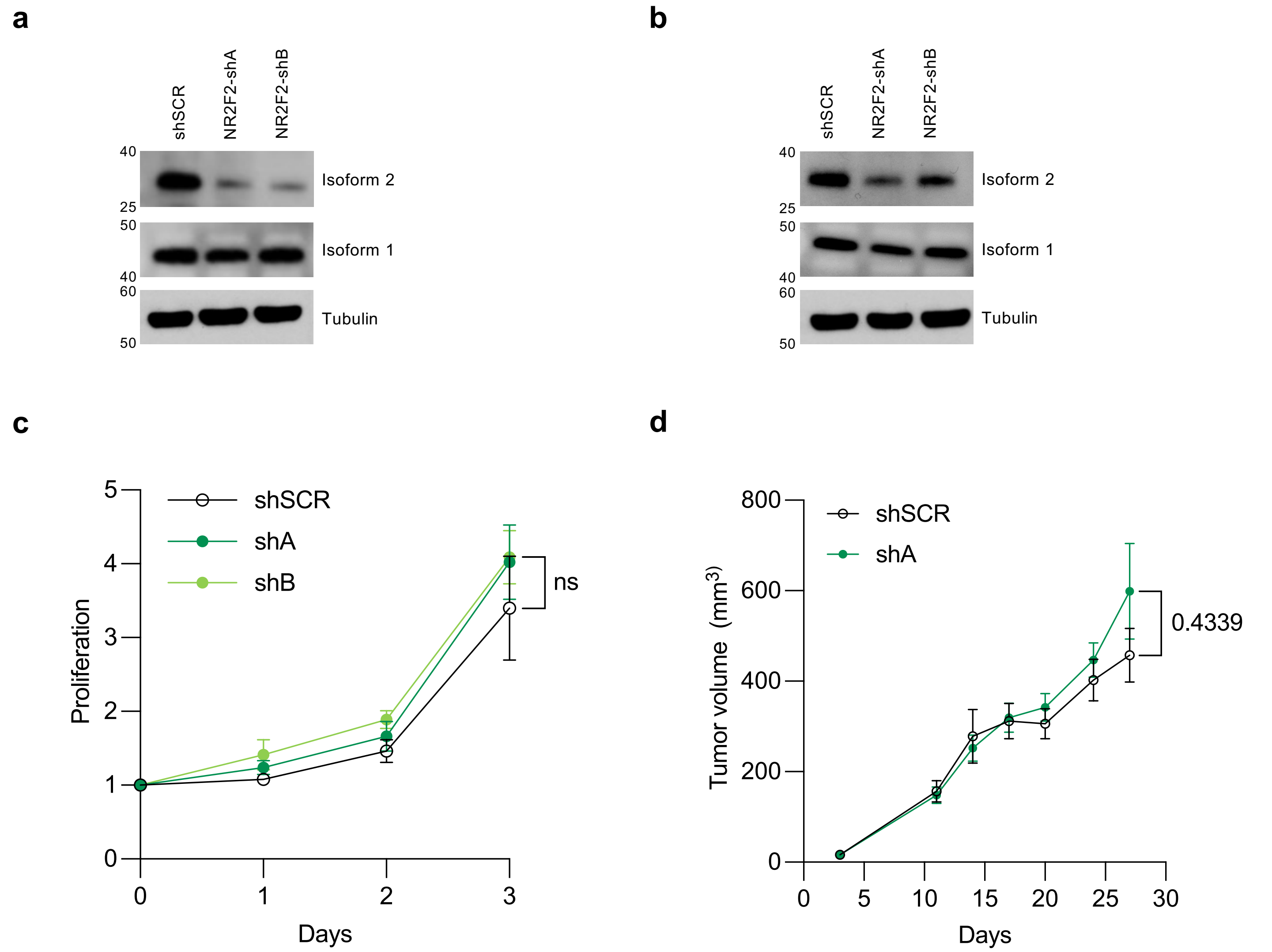

Suppl. Fig 8. NR2F2-Iso2 silencing does not impact proliferation in vitro or in vivo. a. NR2F2 isoform 2 knock-down in 12-273BM (**a**) and WM278 (**b**) melanoma cells confirmed by immunoblot. Tubulin is used as loading control. **c.** Proliferation curve of 12-273BM control (SCR) and cells with silenced Iso2 expression (shA and shB); Two-way ANOVA. **d**. *NR2F2*-Iso2 depletion in 4L cell line did not affect tumor growth by subcutaneous injection in mouse flanks**;** Two-way ANOVA. Bars represent SEM. Experiments have been conducted twice each. Source data are provided as a Source Data file.

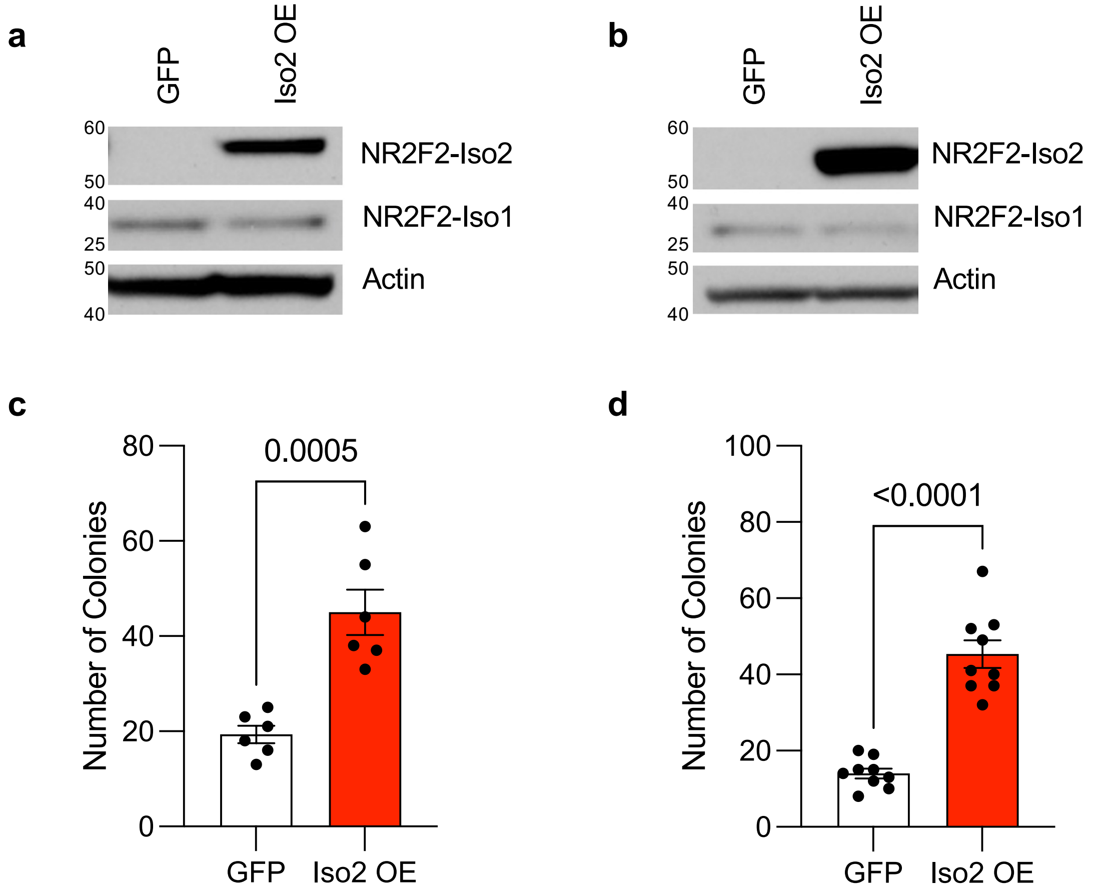

Suppl. Fig 9. Colony formation assays in melanoma cells with *NR2F2*-Iso2 hypermethylation ectopically expressing NR2F2-Iso2 or control GFP. NR2F2-isoform 2 overexpression in IGR-1 (**a**) and SK-MEL-197 (**b**) confirmed by immunoblot. Increased colony formation ability upon NR2F2-Iso2 ectopic expression found in MeWo cells (Fig. 2l) was confirmed in two additional melanoma cell lines: IGR-1 (**c**) and SK-MEL-197, after 21 days (**d**). Unpaired two-tailed T-test. Error bars represent SEM. Experiments have been conducted three times. Source data are provided as a Source Data file.

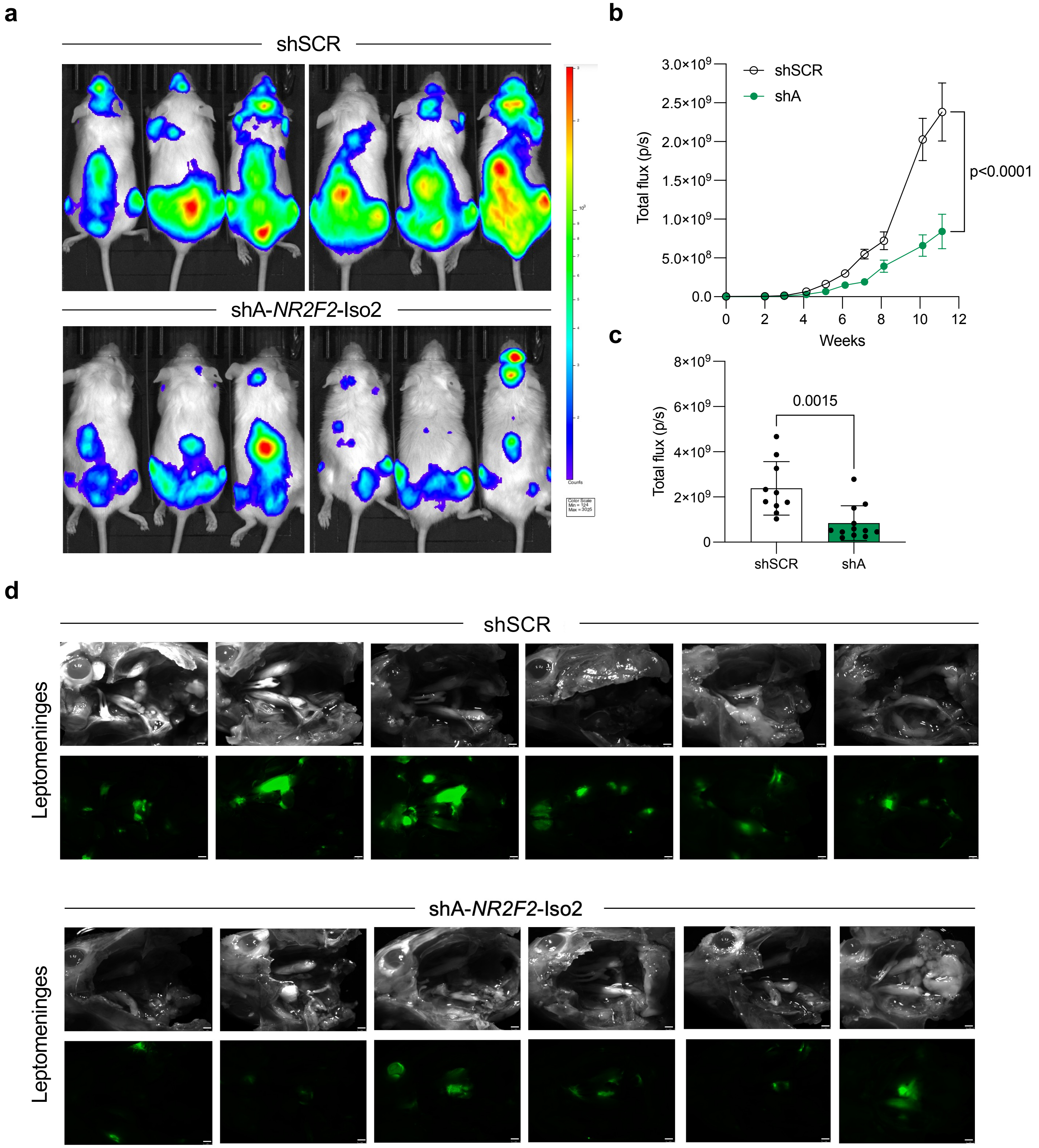

Suppl. Fig. 10. NR2F2 isoform 2 silencing inhibits melanoma metastasis. Loss-of-function (LOF) cellular model was generated in WM278 (melanoma cells, hypo-methylated that express NR2F2-Iso2). Cells were stably transduced with luciferase/mCherry and shA-*NR2F2*-Iso2 or shSCR and GFP to generate NR2F2 isoform 2 loss-of-function cellular systems. **a-d.** WM278 NR2F2-Iso2 depleted (sh*NR2F2*-Iso2) or control (SCR) cells were injected intracardially into NSG mice (n=10 shSCR, 12 shA). A significant reduction in metastatic ability was detected in shA-*NR2F2*-Iso2 group (**a-c**). Six representative mice (**a**) and corresponding leptomeningeal metastases (*ex vivo* GFP fluorescence microcopy images, right panel) from one independent experiment is shown (**d**). Two-way ANOVA, error bars represent standard error of the mean (**b**). Unpaired two-tailed T-test, error bars represent SEM (**c**). Scale bars=1cm in macroscopic images of leptomeninges. Source data are provided as a Source Data file.

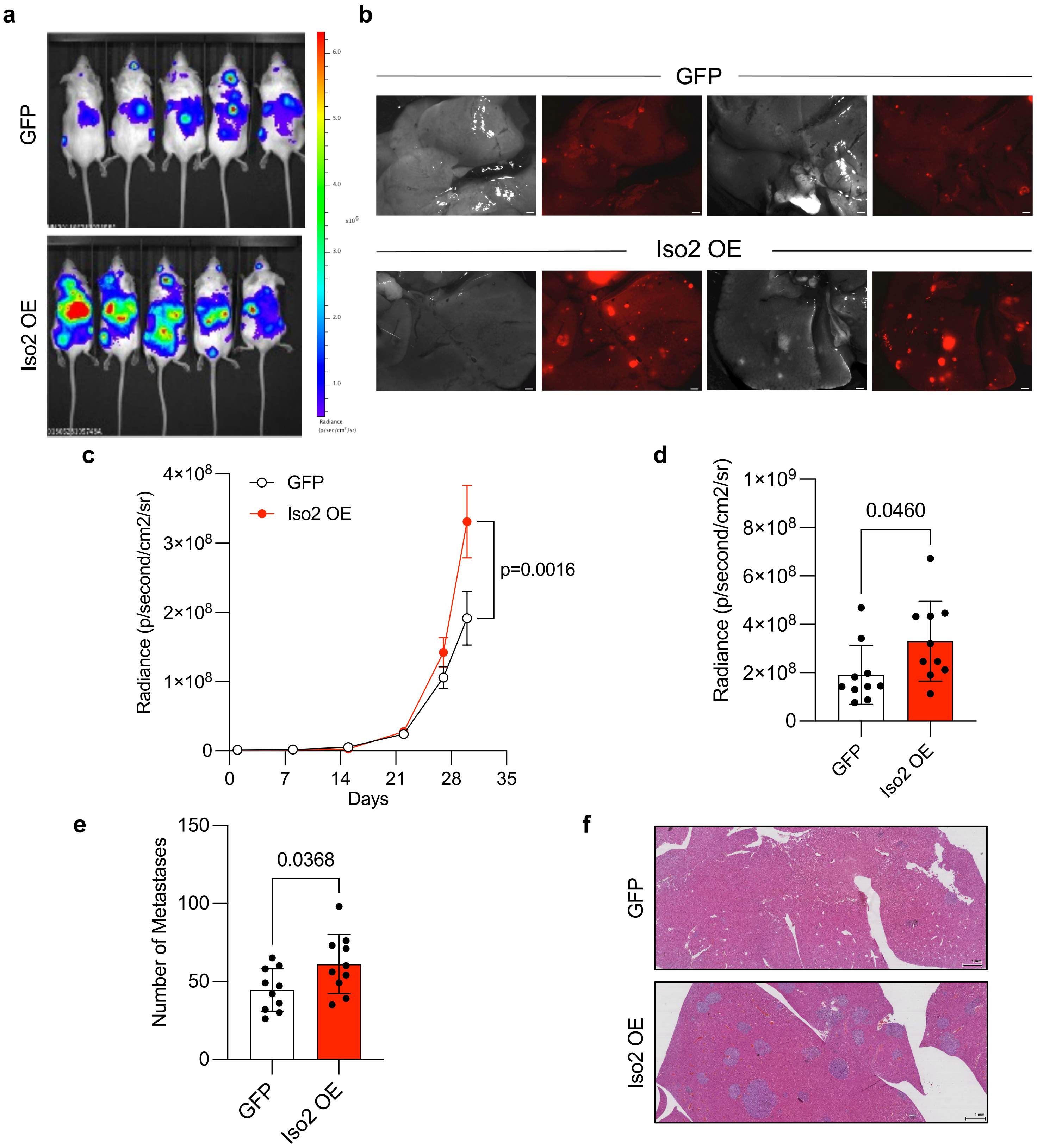

Suppl. Fig. 11. NR2F2 isoform 2 promotes melanoma metastasis. Gain-of-function (GOF) cellular model was generated in MeWo (melanoma cells with hypermethylated and silenced NR2F2-Iso2) ectopically expressing isoform 2 (Iso2). Cells were intracardiac injected into NSG mice (n=10 per group). **(a,b)**. Representative luciferase images (a) and *ex vivo* fluorescence microscopy of dissected livers (b) showing a significant increase in melanoma metastatic potential upon Iso2 ectopic expression from one independent experiment is shown. Radiance levels, monitored over time (**c**, two-way ANOVA, bars represent SEM) and at the end of the experiment (**d**, unpaired two-tailed T-test, bars represent SD), show significant differences between groups. Number of metastatic foci per liver section (**e**, unpaired two-tailed T-test, bars represent SEM) with representative images of H&E stained sections (**f**). Scale bars=1cm in macroscopic images of livers, 1mm in histological sections. Source data are provided as a Source Data file.

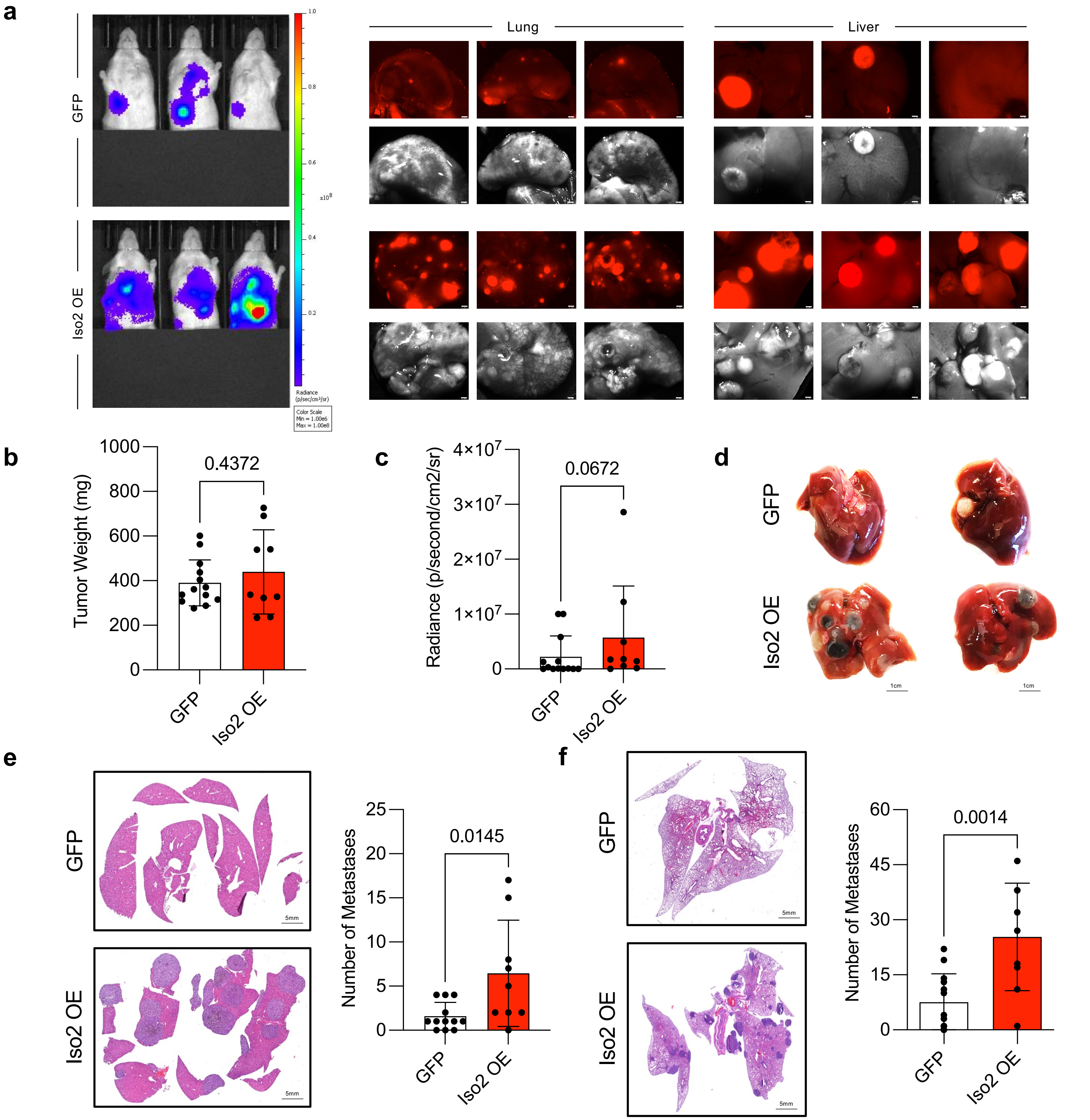

Suppl. Fig. 12. NR2F2 isoform 2 promotes melanoma metastasis. *In vivo* model generated by subcutaneous injection in mice. MeWo (hypermethylated and silenced NR2F2-Iso2) stably transduced with empty or NR2F2-iso2 expressing vectors were subcutaneously injected in mice (control: n=12, Iso2 OE: n=9). After surgical resection of primary tumors, metastasis was assessed. Representative luciferase images (left) and *ex vivo* fluorescence microscopy of dissected livers (right) show a significant increase in melanoma metastatic potential upon Iso2 ectopic expression, one independent experiment is shown (**a**). Weight of subcutaneous tumors at the time of resection did not show differences among groups (**b,** unpaired two-tailed T-test)**.** Radiance levels at endpoint show an increase in metastatic burden upon iso2 ectopic expression (**c**, two-tailed Mann Whitney test). Representative gross images of livers (**d**) and corresponding H&E stained sections and number of metastatic foci per liver section (**e,** unpaired two-tailed T-test). Number of metastatic foci per lung section and representative images of H&E stained sections (**f**). Scale bars: 5mm in histological sections, 1cm in liver and lung macroscopic pictures. Error bars represent standard deviation. Source data are provided as a Source Data file.

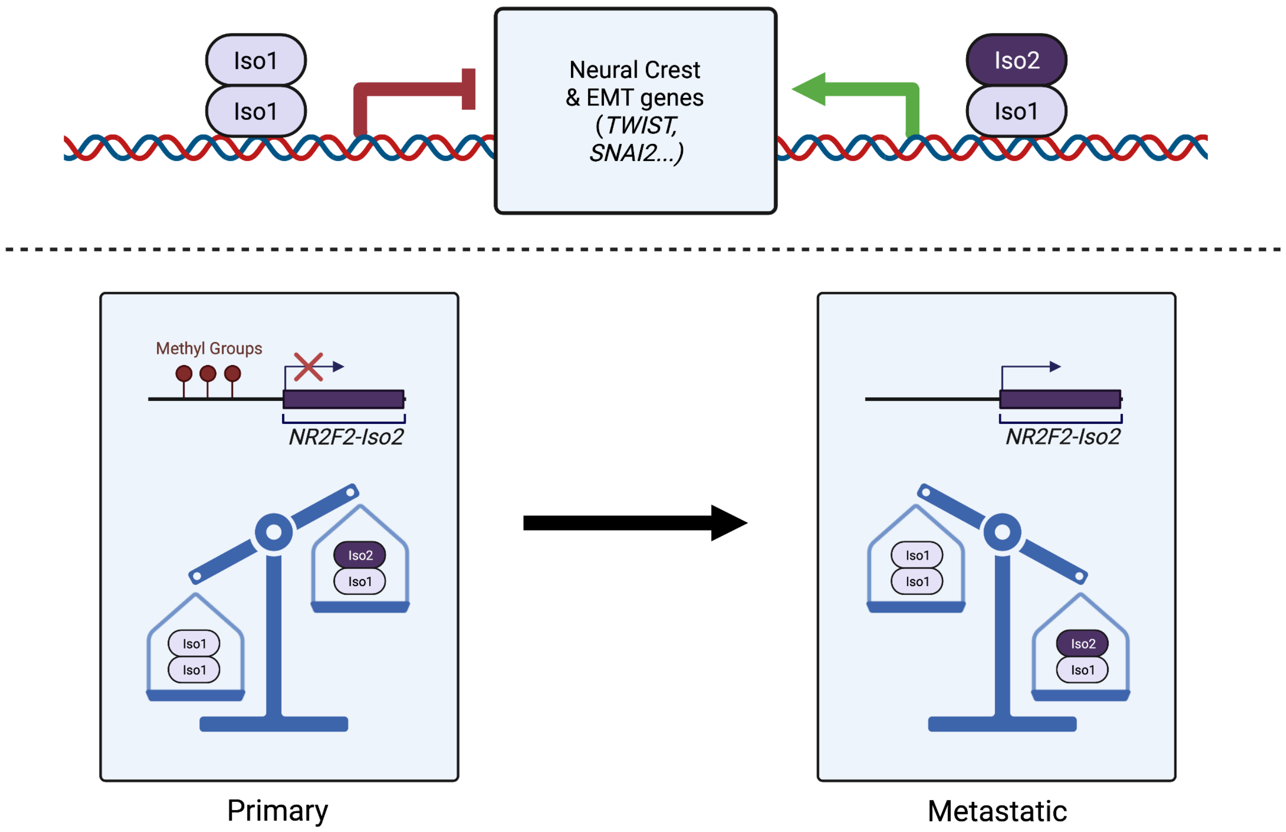

Suppl. Fig. 13. Proposed model of NR2F2-mediated melanoma pathogenesis. Hypermethylation of the *NR2F2-iso2* locus prevents iso2 transcription and translation, yielding a greater level of iso1 homodimers in primary melanoma cells *(bottom)*, inhibiting the transcription of neural crest and EMT genes *(top)*. Hypomethylation of the *NR2F2-iso2* locus allows for iso2 transcription and subsequent translation, yielding a greater level of iso1-iso2 heterodimers in metastatic melanoma cells *(bottom)*, promoting the transcription of neural crest and EMT genes (*top*). Figure was created with Biorender.com.
