# Supplementary material for: An epigenetic switch controls an alternative NR2F2 isoform that unleashes a metastatic program in melanoma
Source: Nat Commun. 2023 Apr 4;14:1867. doi: 10.1038/s41467-023-36967-2 (PMC10073109; doi:10.1038/s41467-023-36967-2)
Supplement: Supplementary file 2 — Description of Additional Supplementary Files [file 41467_2023_36967_MOESM2_ESM.pdf]

Description of Additional Supplementary Files

File Name: Supplementary Data 1
Description: **NR2F2-Iso2 signature gene table.** Data table showing genes consistently down-regulated in shNR2F2-Iso2 compared to shSCR in 4L and 12-273BM melanoma cells.

File Name: Supplementary Data 2
Description: **NR2F2 ChIP-seq table.** Data tables showing (bed NR2F2-like motifs combined) location of NR2F2 ChIP-seq peaks (+/- 100bp from peak summit) with NR2F2-like motifs in 4L cells we identified with MEME-ChIP, (GREAT) prediction of NR2F2 target genes with GREAT, (VENN) intersection of GREAT predicted NR2F2 target genes with mRNA up- or down-regulated after shNR2F2-iso knock-down, (DAVID GO up-chip) DAVID-Gene Ontology of GREAT predicted NR2F2 target genes that increased after NR2F2-iso2 knock-down, (DAVID GO down-chip) DAVID-Gene Ontology of GREAT predicted NR2F2 target genes that decreased after NR2F2-iso2 knock-down, (DAVID GO top 3000 DGE) DAVID-Gene Ontology of the 3000 most differentially expressed and GREAT predicted NR2F2 target genes after NR2F2-iso2 knock-down.
