# Supplementary material for: An epigenetic switch controls an alternative NR2F2 isoform that unleashes a metastatic program in melanoma
Source: Nat Commun. 2023 Apr 4;14:1867. doi: 10.1038/s41467-023-36967-2 (PMC10073109; doi:10.1038/s41467-023-36967-2)
Supplement: Supplementary file 5 — Reporting Summary [file 41467_2023_36967_MOESM5_ESM.pdf]

nature research

Corresponding author(s): Eva Hernando and Veronica Davalos
Last updated by author(s): 01/14/2023

Reporting Summary

Nature Research wishes to improve the reproducibility of the work that we publish. This form provides structure for consistency and transparency in reporting. For further information on Nature Research policies, see our Editorial Policies and the Editorial Policy Checklist.

Statistics

For all statistical analyses, confirm that the following items are present in the figure legend, table legend, main text, or Methods section.

n/a	Confirmed	
☐	☒	The exact sample size (*n*) for each experimental group/condition, given as a discrete number and unit of measurement
☐	☒	A statement on whether measurements were taken from distinct samples or whether the same sample was measured repeatedly
☐	☒	The statistical test(s) used AND whether they are one- or two-sided<br>*Only common tests should be described solely by name; describe more complex techniques in the Methods section.*
☒	☐	A description of all covariates tested
☐	☒	A description of any assumptions or corrections, such as tests of normality and adjustment for multiple comparisons
☐	☒	A full description of the statistical parameters including central tendency (e.g. means) or other basic estimates (e.g. regression coefficient) AND variation (e.g. standard deviation) or associated estimates of uncertainty (e.g. confidence intervals)
☐	☒	For null hypothesis testing, the test statistic (e.g. F, t, r) with confidence intervals, effect sizes, degrees of freedom and P value noted<br>*Give P values as exact values whenever suitable.*
☒	☐	For Bayesian analysis, information on the choice of priors and Markov chain Monte Carlo settings
☒	☐	For hierarchical and complex designs, identification of the appropriate level for tests and full reporting of outcomes
☐	☒	Estimates of effect sizes (e.g. Cohen's *d*, Pearson's *r*), indicating how they were calculated

Our web collection on statistics for biologists contains articles on many of the points above.

Software and code

Policy information about availability of computer code

Data collection

Data analysis

For RNA-seq, sequencing results were demultiplexed and converted to FASTQ format using Illumina bcl2fastq software. Reads were aligned to the human genome (build hg19/GRCh37) with Bowtie 261 using local alignment. Duplicate reads were discarded using Picard [http://broadinstitute.github.io/picard/]. MACS62 was utilized to perform broad peak calling for each replicate with a q-value cutoff of 0.01. Bedtools 63 was used to identify peaks that were called in either SCR, shA, or both conditions and their binding profiles were visualized as histograms using Deeptools64. Regions of maximum central enrichment in ChIPseq peaks were identified by CentriMo65 (MEME-suite66,67). Genes associated with NR2F2-ChIP-seq peaks were identified with GREAT - Genomic Regions Enrichment of Annotations Tool35 - using standard parameters. Differentially enriched transcription factor motifs on NR2F2-bound sites that are up- or down-regulated dependent on NR2F2-iso2 expression were discovered with HOMER38 motif analyses tools. Venn diagrams were generated with Biovenn.nl68. For DNA methylation profile analyses, we used BioEdit software and methylated cytosines were mapped using BSMap software. Fluorescent signal from the microarray was measured with a HiScan scanner (Illumina, Inc. San Diego) using iScan Control Software (V 3.3.29). We did not create custom algorithms.

For manuscripts utilizing custom algorithms or software that are central to the research but not yet described in published literature, software must be made available to editors and reviewers. We strongly encourage code deposition in a community repository (e.g. GitHub). See the Nature Research guidelines for submitting code & software for further information.

Provide a description of all commercial, open source and custom code used to analyse the data in this study, specifying the version used OR state that no software was used.

Data

Policy information about availability of data

All manuscripts must include a data availability statement. This statement should provide the following information, where applicable:

- Accession codes, unique identifiers, or web links for publicly available datasets
- A list of figures that have associated raw data
- A description of any restrictions on data availability

Data is deposited in SuperSeries GSE102554, which includes: RNA-seq (GSE102553) and ChIP-seq (GSE102552). Session is currently private until publication
You can access it here:
https://www.ncbi.nlm.nih.gov/geo/query/acc.cgi?acc=GSE102554
The submission is currently private.
You and the reviewers can use the following secure token to review of record GSE102554 while it remains in private status: qzmrqqmoprsxxyb. It will be open to general access upon notice of publication.
Other datasets analyzed are SKCM TGCA, GSE120878, etc.

Field-specific reporting

Please select the one below that is the best fit for your research. If you are not sure, read the appropriate sections before making your selection.

☒ Life sciences ☐ Behavioural & social sciences ☐ Ecological, evolutionary & environmental sciences

For a reference copy of the document with all sections, see nature.com/documents/nr-reporting-summary-flat.pdf

Life sciences study design

All studies must disclose on these points even when the disclosure is negative.

Sample size	Power calculations were conducted
Data exclusions	There were no data points excluded
Replication	All in vitro experiments were conducted with biological and technical triplicates. All in vivo experiments contained groups with at least 10 mice, where none were excluded from final analysis. All replicated experiments were consistent. Multiple cell lines were used for both gain-of-function and loss-of-function experiments.
Randomization	Mice were randomized before injecting them with melanoma cells. We made sure all mice in each group had the same age and groups had same average weight at the time of injection.
Blinding	Histological samples were provided to a pathologist to estimate metastasis burden. He was blinded to the groups. The investigators were blinded to group allocation during data collection and/or analysis. I

Reporting for specific materials, systems and methods

We require information from authors about some types of materials, experimental systems and methods used in many studies. Here, indicate whether each material, system or method listed is relevant to your study. If you are not sure if a list item applies to your research, read the appropriate section before selecting a response.

Materials & experimental systems

n/a	Involved in the study	
☐	☒	Antibodies
☐	☒	Eukaryotic cell lines
☒	☐	Palaeontology and archaeology
☐	☒	Animals and other organisms
☒	☐	Human research participants
X		Clinical data
☒	☐	Dual use research of concern

Methods

n/a	Involved in the study	
☐	☒	ChIP-seq
☐	☒	Flow cytometry
☒	☐	MRI-based neuroimaging

Antibodies

Antibodies used	NR2F2 Isoform 1 (41859, Abcam for western blot; 61214, Active Motif for ChIP), NR2F2 Isoform 2 (Millipore custom antibody ABE2586), Actin (A3854, Sigma), Lamin-B (sc6217, Santa Cruz), Alpha-tubulin (T9026, Sigma). SNAIL (3879, Cell Signaling Technology). Dilutions indicated in the manuscript. Rabbit secondary (Sigma, A0545; 1:20,000); mouse secondary (Sigma, A9044; 1,20,000); rat secondary (Millipore, AP136P; 1,20,000; and goat secondary (Sigma, A5420; 1,20,000).
Validation	Total protein was extracted using RIPA buffer (Pierce) with protease inhibitors (Roche) and phosphatase inhibitors (Roche). Cell lysates were resolved on NuPAGE 4-12% Bis-Tris Gels (Invitrogen) and transferred to PVDF membranes (Millipore). Membranes were blocked for 1 hour with 5% Blotting Grade Blocker (Bio-Rad) and probed with primary antibodies overnight at 4°C. Membranes were then probed with peroxidase conjugated secondary antibodies.<br>Abcam 41859: Mouse monoclonal to NR2F2; Suitable for: IHC-P, WB; Reacts with: Mouse, Rat, Human; Isotype: IgG2a<br>Active Motif 61214: Polyclonal; Isotype: IgG; Affinity Purified; Host: Rabbit; Reactivity: Human<br>Millipore ABE2586: rabbit polyclonal antibody that targets NR2F2 and has been tested for use in Western Blotting.<br>Snail (C15D3) Rabbit mAb #3879. Species Reactivity: human, mouse, rabbit, monkey. Source/purification: monoclonal antibody is produced by immunizing animals with a recombinant human Snail protein. Snail (C15D3) Rabbit mAb detects endogenous levels of total Snail protein. Validation of antibodies was conducted by using lysates of cells with knock-down, knock-out or ectopic overexpression of the protein of interest.

Eukaryotic cell lines

Policy information about cell lines

Cell line source(s)	The H9 embryonic stem cell line was purchased from WiCell Institute and maintained in co-culture with MitC-treated primary mouse embryonic fibroblasts (MEFs) in the presence of FGF2 (6 ng/ml; R&D Systems), under conditions described by the supplier. Neural crest-derived primary cell lines (NCCs) were isolated in accordance with institutional authorities' guidelines and French legal regulations (Bioethics law 2004-800 and Protocol PFS14-011), as previously described. NCC1 (90003), NCC2

(SZ08) and NCC3 (SZ15) were derived from 7 post-conceptional week (PCW) dorsal root ganglia (DRG), while NCC4 (SZ112) was derived from migratory Schwann cell precursors explanted from an 11 PCW brachial plexus. All NCCs were grown in collagen I (BD bioscience)-coated plates using the following medium: Dulbecco's Modified Eagle Medium/Nutrient Mixture F-12 with GlutaMAX supplemented with 12% embryonic stem cell qualified fetal bovine serum (ATCC), 1% penicillin/streptomycin (HyClone); 10 mM HEPES (Invitrogen), 0.1 ug/ml hydrocortisone (Sigma-Aldrich), 10 ug/ml transferrin (Sigma-Aldrich), 0.4 ng/ml T3 (3,3,5-thio-iodo-thyronine) (Sigma-Aldrich), 10 pg/ml glucagon (Sigma-Aldrich), 1 ng/ml insulin (Sigma-Aldrich), 100 pg/ml epidermal growth factor (Sigma Aldrich), and 200 pg/ml fibroblast growth factor 2 (Gibco)41.
Human Epidermal Melanocytes (HEMs) isolated from neonatal human skin were purchased from ScienCell Research Laboratories. HEMs were grown in poly-L-lysine-coated plates using MelM melanocyte medium (Cat. #2201, ScienCell Research Laboratories), as recommended by the supplier. Human cell lines were acquired as follows: 501mel from Yale University; 293T, A-375, 451Lu, MeWo, SK-MEL-2, and IGR-1 from American Type Culture Collection (ATCC); SK-MEL-85, SK-MEL-100, SK-MEL-103, SK-MEL-147, SK-MEL-197 were kindly provided by Alan Houghton (Memorial Sloan–Kettering Cancer Center, New York, NY, USA); WM278 from Meenhard Herlyn (Wistar Institute, Philadelphia, PA, USA) and WM239-derived 113/6-4L (designed as 4L) from Robert S Kerbel and William Cruz-Munoz26 (Sunnybrook Research Institute, Toronto, Canada); low passage melanoma short-term cultures (STCs), including 12-273BM, 10-230SC and 12-126BM were derived in Dr. Iman Osman laboratory as described (PMID: 22404973 ; PMID: 35262173) and grown in DMEM with 10% fetal bovine serum (FBS), 1mM Sodium Pyruvate, 4mM L-Glutamine, 25 mM D-Glucose, 1% Non-essential Amino Acids (NEAA), 100units/mL penicillin, and 100mg/mL streptomycin.

Authentication

Cell lines were validated using the STR method at ATCC. All STCs were matched to the respective donor (De Miera, PCMR, 2019). The identity of non-ATCC cells was validated using Promega's Cell ID system (Cat. # G9500) by STR analysis.

Mycoplasma contamination

All cell lines used in the study were tested negative for Mycoplasma contamination prior to use in experiments. None were found contaminated.

Commonly misidentified lines
(See ICLAC register)

No commonly misidentified cell lines were used in the study.

Animals and other organisms

Policy information about studies involving animals; ARRIVE guidelines recommended for reporting animal research

Laboratory animals

NOD.Cg-Prkdcscid Il2rgtm1Wjl/SzJ (NSG) mice (The Jackson Laboratory) - male, 6-8 weeks old. Athymic nude male mice (Jackson labs, Cat # 002019); 6-8 weeks old. We have included information on housing conditions for the mice, describing dark/light cycle, ambient temperature and humidity in the manuscript.

Wild animals

Study did not involve wild animals.

Field-collected samples

Study did not involve samples collected from the field.

Ethics oversight

Animal experiments were conducted in accordance with guidelines set forth by the Institutional Animal Care and Use Committee (IACUC) of NYU (protocol # 120405-02)

Note that full information on the approval of the study protocol must also be provided in the manuscript.

Clinical data

Policy information about clinical studies
All manuscripts should comply with the ICMJE guidelines for publication of clinical research and a completed CONSORT checklist must be included with all submissions.

Clinical trial registration

Provide the trial registration number from ClinicalTrials.gov or an equivalent agency.

Study protocol

Note where the full trial protocol can be accessed OR if not available, explain why.

Data collection

Describe the settings and locales of data collection, noting the time periods of recruitment and data collection.

Outcomes

Describe how you pre-defined primary and secondary outcome measures and how you assessed these measures.

ChIP-seq

Data deposition

☒ Confirm that both raw and final processed data have been deposited in a public database such as GEO.

☒ Confirm that you have deposited or provided access to graph files (e.g. BED files) for the called peaks.

Data access links
May remain private before publication.

https://www.ncbi.nlm.nih.gov/geo/query/acc.cgi?acc=GSE102554
The submission is currently private.
You and the reviewers can use the following secure token to review of record GSE102554 while it remains in private status: qzmrqqmoprsxxyb

Files in database submission

GSM2740590 4L-SCR_Input
GSM2740591 4L-SCR_NR2F2 ChIPseq
GSM2740592 4L-shIso2_Input
GSM2740593 4L-shIso2_NR2F2 ChIPseq

Genome browser session
(e.g. UCSC)

No longer applicable

Methodology

Replicates	2 replicates; replicate A: 5386, replicate B: 2544; replicate A and B 2208.
Sequencing depth	All experiments were single end sequenced, 50 bp. The number of total reads varies from 30M to 120M reads. The number of unique peaks varied from 2,524 to 5,386.
Antibodies	ChIP grade NR2F2 antibody specific for isoform 1 (catalog # 61214, Active Motif)
Peak calling parameters	callpeak --format BAM --bdg --verbose 2 --qvalue 0.01 --gsize hs --trackline
Data quality	qvalue cutoff = 1.00e-02; 5386 peaks are called; 4385 peaks contain NR2F2-like motif; 3315 peaks are more than 5-fold enriched.
Software	MACS version 2.1.0.20140616

Flow Cytometry

Plots

Confirm that:

- [ ] The axis labels state the marker and fluorochrome used (e.g. CD4-FITC).
- [ ] The axis scales are clearly visible. Include numbers along axes only for bottom left plot of group (a 'group' is an analysis of identical markers).
- [ ] All plots are contour plots with outliers or pseudocolor plots.
- [ ] A numerical value for number of cells or percentage (with statistics) is provided.

Methodology

Sample preparation	*Describe the sample preparation, detailing the biological source of the cells and any tissue processing steps used.*
Instrument	*Identify the instrument used for data collection, specifying make and model number.*
Software	*Describe the software used to collect and analyze the flow cytometry data. For custom code that has been deposited into a community repository, provide accession details.*
Cell population abundance	*Describe the abundance of the relevant cell populations within post-sort fractions, providing details on the purity of the samples and how it was determined.*
Gating strategy	*Describe the gating strategy used for all relevant experiments, specifying the preliminary FSC/SSC gates of the starting cell population, indicating where boundaries between "positive" and "negative" staining cell populations are defined.*

- [ ] Tick this box to confirm that a figure exemplifying the gating strategy is provided in the Supplementary Information.
